# Tissue-specific autoantibody signatures reveal immune alterations undetected by routine serology in long COVID

**DOI:** 10.1007/s11357-026-02286-9

**Published:** 2026-05-12

**Authors:** Ottó Tatai, Szilárd Nagy, Trai Huynh Thanh Nguyen, Beáta Lajszné Tóth, Péter Antal-Szalmás, Ivetta Mányiné Siket, Tamás Bence Pintér, Miklós Fagyas, Zoltán Papp, Péter Csécsei, Andrea Lehoczki, Ágnes Szappanos, Zoltan Ungvari, Tihamér Molnár, Attila Tóth

**Affiliations:** 1 Division of Clinical Physiology, Department of Cardiology, Faculty of Medicine, University of Debrecen, Debrecen, Hungary; 2 Department of Laboratory Medicine, Faculty of Medicine, University of Debrecen, Debrecen, Hungary; 3 Department of Neurosurgery, Medical School, University of Pecs, Ret U 2, 7624 Pecs, Hungary; 4 Institute of Preventive Medicine and Public Health, Semmelweis University, Budapest, Hungary; 5 Fodor Center for Prevention and Healthy Aging, Semmelweis University, Budapest, Hungary; 6 Institute for Translational Research, Budapest, Hungary; 7 Heart and Vascular Center, Semmelweis University, Budapest, Hungary; 8 Department of Rheumatology and Immunology, Semmelweis University, Budapest, Hungary; 9 Vascular Cognitive Impairment, Neurodegeneration, and Healthy Brain Aging Program, Department of Neurosurgery, University of Oklahoma Health Sciences Center, Oklahoma City, OK USA; 10 Doctoral College/Institute of Preventive Medicine and Public Health, International Training Program in Geroscience, Semmelweis University, Budapest, Hungary; 11 Department of Anaesthesiology and Intensive Care, Medical School, University of Pecs, Pecs, Hungary

**Keywords:** Long COVID, Autoimmunity, tissue-specific autoantibodies, autoantibodies, Vascular dysfunction, Microvasculature, Immunosenescence, Immune dysregulation, Western blot, Cardiovascular system, Neurovascular symptoms, Serological diagnostic, Biomarkers

## Abstract

Long COVID affects a substantial proportion of individuals recovering from severe acute respiratory syndrome coronavirus 2 (SARS-CoV-2) infection, yet its underlying pathophysiology remains poorly understood. Although autoimmunity is increasingly implicated in disease pathogenesis, routine diagnostics frequently fail to detect relevant immune dysregulation. To address this gap, we analyzed sera from Long COVID patients (*n* = 114) and pre-pandemic controls (*n* = 36) using tissue-based Western blotting targeting cardiac, pulmonary, and vascular antigens, alongside standard ANA HEp-2 testing. Longitudinal samples were additionally evaluated to assess autoantibody dynamics. Autoantibodies were detected in the majority of patients (83% vs. 53% in controls; *p* < 0.05), showing a dominant cardiovascular pattern. While cardiac (54% vs. 33% in controls; *p* = 0.16) and pulmonary (34% vs. 30% in controls; *p* = 0.50) prevalences did not reach significance, vascular autoreactivity was markedly elevated in Long COVID (34% vs. 8% in controls; *p* < 0.05). Responses exhibited broad polyreactivity and IgM dominance, with longitudinal follow-up showing persistent IgM and the emergence of additional isotypes. Clinically, cardiac autoreactivity was associated with hypertension and headache, while the overall autoreactivity correlated with anosmia and ageusia. In contrast, ANA HEp-2 testing showed no discriminatory value or clinical associations. Distinct autoreactivity patterns further aligned with female sex and clinical parameters (C-reactive protein, creatinine, troponin, body mass index). Together, these findings reveal a high burden of tissue-specific autoantibodies invisible to standard ANA screening. This persistent, IgM-skewed profile suggests ongoing immune dysregulation and may reflect a previously underrecognized component of the immunological response in long COVID, highlighting the need for targeted immunodiagnostic approaches beyond routine serology.

**Supplementary Information:**

The online version contains supplementary material available at 10.1007/s11357-026-02286-9.

## Introduction

The coronavirus disease 2019 (COVID-19) pandemic has been followed by a growing recognition of persistent post-infectious manifestations in recovered individuals, collectively termed post-acute sequelae of COVID-19 or long COVID [1, 2]. While definitions vary globally, this study adopts the criteria proposed by the National Institute for Health and Care Excellence (NICE), which encompass persistent or newly emerging symptoms lasting more than 4 weeks after acute SARS-CoV-2 infection [3]. Current epidemiological estimates suggest a substantial burden; a recent systematic review indicates a global pooled prevalence of approximately 36% among infected individuals, potentially affecting hundreds of millions worldwide [4, 5].

Long COVID is a multifactorial condition with a highly heterogeneous presentation. Several biological mechanisms have been proposed, including persistent viral reservoirs, chronic inflammation, thrombosis, dysbiosis, and mitochondrial dysfunction [6, 7]. Crucially, autoimmunity has emerged as a key mechanism in pathogenesis. Studies have detected functional autoantibodies targeting cardiac, pulmonary, and nuclear antigens in patients with severe acute COVID-19, often correlating with disease severity [8–10]. Notably, many of these autoantibodies mirror those found in chronic autoimmune diseases such as systemic lupus erythematosus and rheumatoid arthritis [11–14]. However, while the autoimmune landscape of acute infection is increasingly characterized, the extent to which these autoimmune responses persist and drive the chronic symptomatology of long COVID remains less understood.

Clinically, long COVID can affect virtually any organ system, yet cardiovascular and pulmonary sequelae are particularly prominent and debilitating. Cardiovascular manifestations frequently include chest pain, palpitations, and arrhythmias, with potential complications extending to myocardial inflammation and autonomic dysfunction (e.g., POTS) [15–18]. The underlying pathophysiology likely involves a synergy of direct viral toxicity, ACE2 downregulation, and immune-mediated injury [15]. Pulmonary involvement is dominated by dyspnea and cough, often accompanied by radiological findings of ground-glass opacities or fibrosis [17]. These changes are thought to result from sustained endothelial-epithelial barrier disruption and perivascular inflammation [19]. Beyond the cardiopulmonary system, the syndrome manifests with a broad spectrum of neuropsychiatric (e.g., cognitive decline, anxiety), thromboembolic, renal, gastrointestinal, endocrine, and dermatological abnormalities [15, 20–24].

Despite this high symptom burden, the current management of long COVID remains largely supportive and symptom-oriented. Therapeutic guidelines, such as those from NICE, prioritize holistic rehabilitation and the exclusion of alternative diagnoses, but lack targeted, disease-modifying interventions [3, 25]. While corticosteroids, anticoagulants, and cognitive behavioral therapy may offer symptomatic relief for specific subsets of patients [26–28], causal therapies are currently lacking due to an incomplete understanding of the underlying pathophysiology. Specifically, in the context of cardiovascular and pulmonary manifestations, emerging evidence indicates that SARS-CoV-2 induces injury not only through direct viral cytotoxicity but also via autoimmune mechanisms, contributing to sustained organ involvement [29].

Our research aims to investigate the potential role of autoimmunity in long COVID patients by detecting autoantibodies targeting cardiac, vascular, and pulmonary antigens and assessing the clinical sequelae associated with these autoimmune responses.

## Methods

### Patient recruitment and blood sampling

The study cohort comprised 114 individuals. Serum samples were obtained at the University of Pécs Clinical Center, Hungary, through a collaborative network with general practitioners (GPs) within the area. Patients exhibiting persistent long COVID symptoms were referred by their primary care physicians to the outpatient clinic of the Department of Anaesthesiology and Intensive Care for baseline evaluation. Inclusion criteria were defined as follows: (i) age ≥ 18 years; (ii) SARS-CoV-2 infection confirmed by positive PCR or antigen test; (iii) a latency of at least 30 days between symptom onset and the outpatient visit; and (iv) persistence of symptoms at the time of presentation. Exclusion criteria comprised pre-existing malignancies, active autoimmune disorders, concurrent immunosuppressive therapy, and acute coronary syndrome. To serve as negative controls, the study also utilized 36 historical serum samples obtained before the emergence of SARS-CoV-2.

Peripheral blood sampling for laboratory analysis was performed at the Department of Anaesthesiology and Intensive Care (University of Pécs) during the initial visit. For a patient subset (*n* = 30), follow-up sampling was conducted after a mean period of 141 days. Patient demographics, clinical history, vaccination status, and comorbidities were obtained via questionnaires and medical records. Samples were maintained at − 70 °C before being transported to the University of Debrecen (Department of Cardiology, Division of Clinical Physiology and Department of Laboratory Medicine) for processing.

### Ethical statement

The study protocol was approved by the Scientific and Research Ethics Committee of the University of Debrecen and the Ministry of Human Capacities (registration number for Long COVID recruitment: IV/2505–3/2021/EKU). The utilization of retrospective human tissue samples was performed under separate ethical permits: 323–82,005—1018/EKU for heart and vascular tissues, 15,471—6/2023/EÜIG for lung tissues, and 33,327—1/2015/EKU for the negative control cohort.

### Detection of tissue-specific autoantibodies using Western blot

A Western blot-based assay was employed to detect autoantibodies using human cardiac, pulmonary, and vascular tissue homogenates as antigen substrates. All Western blot analyses were performed at the Department of Cardiology, Division of Clinical Physiology at the University of Debrecen. Cardiac samples were retrieved from unused donor hearts obtained from multi-organ donors. Healthy pulmonary tissue was isolated from resected specimens during lung cancer surgery, while vascular homogenates were prepared from discarded segments of the internal mammary artery harvested during coronary artery bypass grafting. All samples were kept at − 70 °C until processing. Approximately 50–80 mg of wet tissue was homogenized in SDS-PAGE sample buffer (2 × Laemmli Sample Buffer, Merck KGaA, Darmstadt, Germany) using a Potter homogenizer. The homogenates were boiled for 5 min at 100 °C and centrifuged at 10,000 × *g* for 10 min. The resulting supernatants were collected for further analysis. Protein concentration was determined using a dot blot-based method: 1 µl of each sample was spotted onto a nitrocellulose membrane, stained with Coomassie Brilliant Blue (Merck KgaA, Darmstadt, Germany), and quantified against a Bovine Serum Albumin (BSA) standard curve. Image acquisition was performed using an MF-ChemiBIS 3.2 system (DNR Bio-Imaging Systems, Neve Yamin, Israel), and data were analyzed using GelAnalyzer 23.1.1 software [30].

For each sample, 60 µg of tissue homogenate was loaded onto the gel in a final volume of 15 µl. Protein separation was carried out on 10% SDS-PAGE gels using Bio-Rad electrophoresis equipment (Bio-Rad Laboratories, Hercules, CA, USA). The separated proteins were then transferred to 0.45-µm nitrocellulose membranes (Bio-Rad Laboratories). Following protein transfer, membranes were stained with Ponceau S to visually verify transfer efficiency and ensure equal protein loading. Non-specific binding sites were blocked using 5% non-fat dry milk in phosphate-buffered saline containing 0.1% Tween 20 (PBST). Subsequently, membranes were cut into individual strips for each well (15 strips per gel) and placed in separate incubation chambers (the procedure is depicted in the Supplementary Fig. 1 in detail).

Each strip was incubated with patient serum at a dilution of 1:1000. This dilution was empirically determined through optimization experiments aimed at minimizing the detection of low-affinity, nonspecific (“housekeeping”) antibodies. To establish the optimal dilution, a titration series was performed using heart tissue homogenates, testing primary antibody (patient serum) dilutions ranging from 1:15 to 1:5000. Dilutions lower than 1:1000 produced substantial nonspecific background and increased detection of low-affinity antibodies. In contrast, dilutions greater than 1:1000 resulted in reduced sensitivity for several tissue-specific bands (Supplementary Fig. 2).

Alongside patient sera and the negative control, a positive control was included in each Western blot run. This control consisted of a pre-selected sample with sufficiently high signal intensity, displaying a banding pattern distinguishable from the negative control. While heart tissue reactivity was assessed in the entire cohort (*n* = 114), assays regarding lung and vascular tissues were restricted to randomly selected subsets (*n* = 94 and *n* = 73) due to the limited availability of tissue homogenates. Membrane strips were washed three times in PBST (5 min each) and subsequently pooled into a common chamber for secondary antibody incubation. Peroxidase-conjugated goat anti-human IgG (targeting both heavy and light chains [H + L], 1:13,000) or goat anti-human IgM (1:13,000) were used (Merck KGaA). Following the final wash, strips were incubated with enhanced chemiluminescence (ECL) substrate for 1 min (Advansta WesternBright ECL HRP substrate, San Jose, CA, USA). For imaging, the strips were reassembled in the tray of the MF-ChemiBIS 3.2 device to reconstruct the original membrane layout, and digital images were acquired for further evaluation.

### Detection of antinuclear antibodies on HEp-2 cells

Comparative testing for antinuclear antibodies (ANA) was performed on HEp-20—10 cells (EUROPattern, Euroimmun, Lübeck, Germany) according to a protocol for routine clinical autoantibody diagnostics. Following incubation with patient sera, bound autoantibodies were visualized using fluorescein isothiocyanate (FITC)–labeled anti-human IgG and IgM antibodies. Fluorescent patterns were evaluated using computer-aided immunofluorescence microscopy (EUROPattern, Euroimmun, Lübeck, Germany), and two experts classified the captured images according to the International Consensus on ANA Patterns (ICAP). Samples were initially screened at a dilution of 1:80. Those exhibiting positive fluorescence were subsequently tested in serial dilutions (1:320, 1:1280, 1:5120) to determine the end-point titer. In cases where fluorescence intensity remained strong at a lower dilution but was absent at the next standard step, an intermediate titer (e.g., 1:640) was assigned. In accordance with routine diagnostic protocols, samples showing no reactivity at the 1:80 screening threshold were considered negative. In all cases, the tests were performed using known positive and negative controls.

### Determination of the molecular weight of tissue autoantigens

Quantitative analysis of protein bands was performed using GelAnalyzer 23.1.1 software (developed by the Institute of Chemistry, University of Debrecen) [30]. Molecular weights were determined based on the peak maxima of the bell-shaped intensity profiles characteristic of each band. For quantitative assessment, the intensity profiles of each band were integrated, and the area under the curve (AUC) was used as the measure of signal intensity. Signal intensities were normalized to the total cumulative intensity of all detected protein bands within the respective lane (set as 100%); accordingly, the abundance of specific bands was expressed as a percentage of the total lane intensity (detailed information on the method is available in Supplementary Fig. 3). To differentiate specific autoantibody signals from background noise, an outlier analysis was done using GraphPad Prism 10.0. (GraphPad Software, San Diego, CA, USA). We applied the ROUT method with a strict *Q*-value of 1% (False Discovery Rate) to identify bands with significantly elevated intensities compared to the cohort baseline at identical molecular weights. This statistical approach allowed for the isolation of specific autoantigen-driven signals from non-specific background binding, when the apparent molecular masses overlapped. Ultimately, the results were validated by a committee of at least three Western blot experts. Bands were considered positive only if a consensus was reached by all members. The identified autoantibodies were further evaluated in the context of the available clinical data of the long-COVID cohort using GraphPad Prism software.

### Data analysis and statistics

**Fig. 1:**
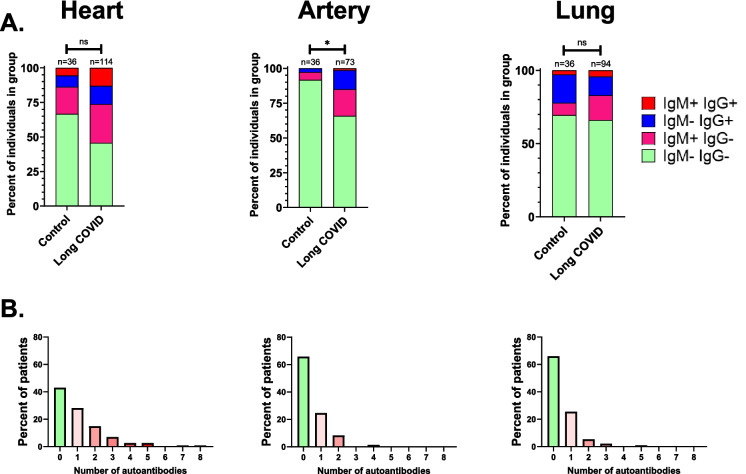
Comparison of tissue-specific autoantibody prevalence between the control and long COVID cohorts. **A** Stacked bar charts illustrate the percentage of patients positive for various autoantibody combinations in heart (*n* = 114), artery (*n* = 73), and lung (*n* = 94) tissues compared to controls (*n* = 36). The color shading indicates the serological profile for each group, where *green* represents samples negative for both IgM and IgG, *magenta* denotes samples positive for IgM only, *blue* represents samples positive for IgG only, and *red* shows positivity for both IgM and IgG. Statistical significance between the Control and Long COVID groups for prevalence was assessed using the chi-square test, with “ns” indicating no significant difference between the cohorts. **B** Histograms display the distribution of the total number of unique autoantibodies detected per patient within each tissue category

Data analyses were performed using GraphPad Prism software 10.0. The chi-square test was applied to the datasets presented in Figs. 1A, 4, and 5. For the variables summarized in Tables 1–5, continuous data were analyzed using the Mann–Whitney test, while categorical data were evaluated using Fisher’s exact test. For Fig. 3, odds ratios (ORs) for categorical variables were calculated using the Baptista–Pike method. For continuous predictors, multiple logistic regression analysis was conducted, and results were standardized to express the odds ratio per interquartile range (IQR).

## Results

Serum samples were used to detect autoantibodies using three different human tissue homogenates as baits (heart, internal mammary artery, and lung). First, the autoantigen-autoantibody cross reactions were detected by a Western blot-based method. We generally found a higher prevalence of autoantibodies recognizing human tissue antigens in the long COVID population when compared to controls (heart, 54% versus 33%, *n* = 114, *p* = 0.16; artery, 34% versus 8%, *n* = 73, *p* < 0.05; lung, 34% versus 31%, *n* = 94, *p* = 0.51, Fig. 1A). Notably, the majority of detected autoantibodies were of the IgM isotype (Fig. 1A). Regarding the binding pattern, approximately half of the autoantibody-positive patients showed reactivity against a single antigen, while the other half exhibited a broad response targeting multiple proteins (up to 8 distinct bands per patient; Fig. 1B).

**Fig. 2:**
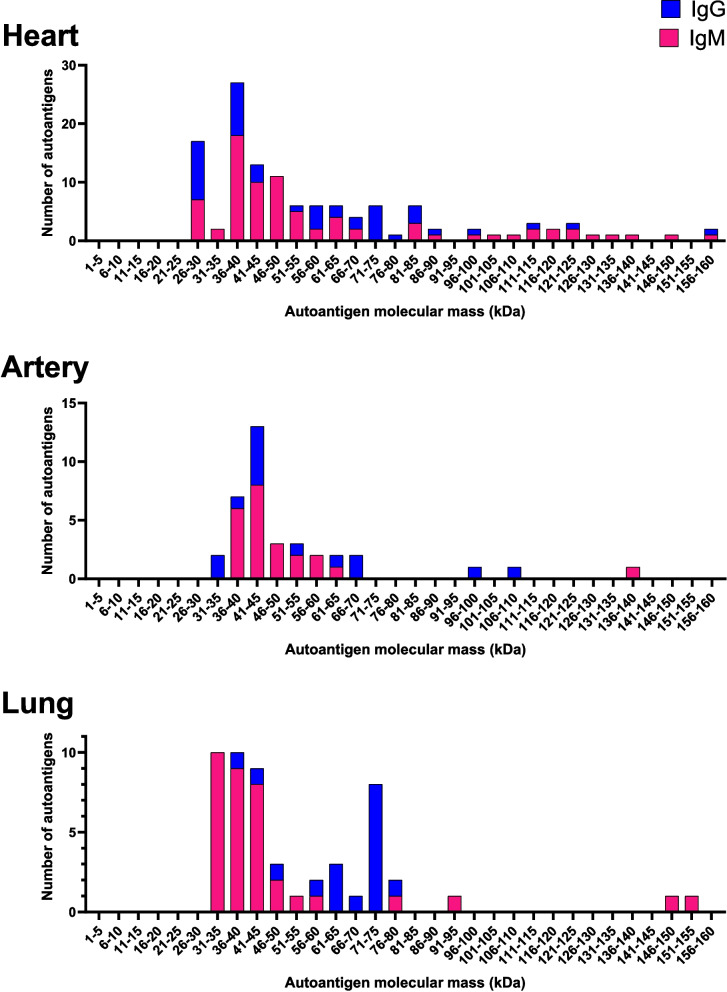
Distribution of detected autoantigen molecular masses across heart, artery, and lung tissues in the long COVID cohort. The *x*-axis represents the molecular mass of autoantigens in kilodaltons (kDa) grouped into 5-kDa increments. The *y*-axis indicates the total number of instances an autoantigen was detected within each mass interval. Stacked bars are color-coded to denote the antibody isotype detected, where *magenta* represents IgM and *blue* represents IgG

The autoantigen distribution did not show a recognizable pattern (Fig. 2). Interestingly, the IgG and IgM isotypes cross-reacted with different antigens in the majority of the cases (Supplementary Fig. 3). This diverse pattern is clearly recognizable in the case of antigens in the lung, where only a limited overlap was seen between IgG and IgM targets, even when evaluated together (Fig. 2). Analysis of molecular weights revealed no specific pattern; the distribution of target antigens simply reflected the expected molecular mass range of proteins expressed in these tissues (Fig. 2).

**Fig. 3:**
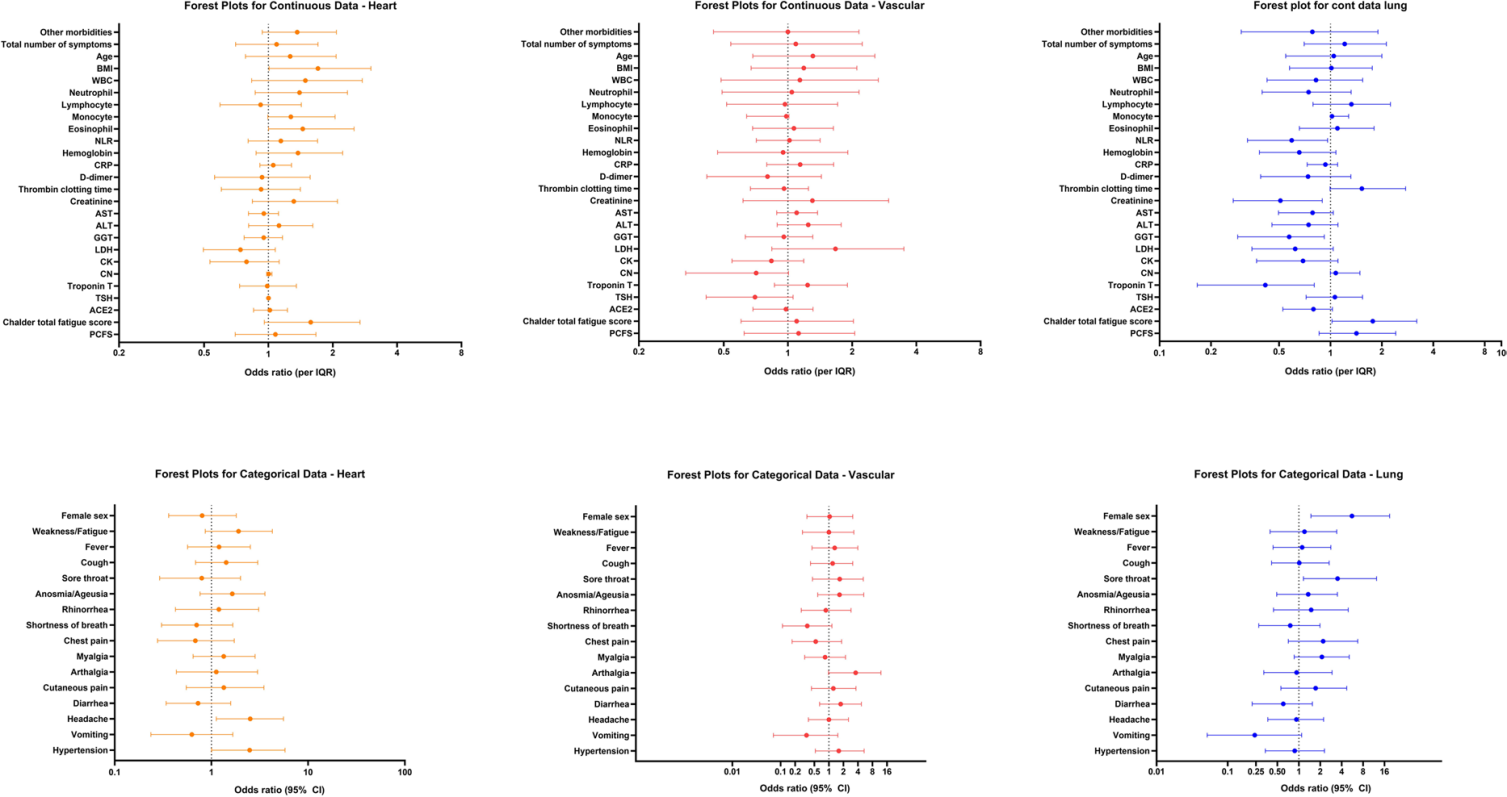
Forest plots demonstrating the association between clinical variables and the presence of autoantibodies for heart, vascular (artery), and lung tissues. The upper sections of each plot display the odds ratios (OR) for continuous variables, including patient demographics and laboratory markers such as CRP, LDH, and d-dimer, calculated per interquartile range (IQR). The lower sections illustrate the odds ratios for categorical variables, including female sex, and Long COVID symptoms such as fever, cough, and shortness of breath. Data is presented with 95% confidence intervals (CI)

Long COVID patients were stratified based on the presence or absence of tissue-specific autoantibodies, and their clinical features were compared (Table 1). While general clinical characteristics were largely similar between the groups, distinct phenotype correlations emerged. Notably, patients positive for anti-cardiac autoantibodies exhibited a significantly higher prevalence of headache (47% vs. 26% in negatives) and hypertension (38% vs. 20%, Table 2 and Fig. 3). Additionally, CRP levels (1.9 vs. 1.2 mg/l) and BMI (27.6 vs. 25.6 kg/m^2^) were slightly elevated in the cardiac autoantibody-positive subgroup (Table 2, Fig. 3). In contrast, no significant differences in clinical parameters were observed between patients testing positive or negative for vascular autoantibodies (Table 3, Fig. 3). Regarding pulmonary autoreactivity, serum creatinine (63 vs. 69 μmol/L) and cardiac troponin T (3.6 vs. 4.0 ng/L) levels were slightly lower in the positive group; however, this is likely attributable to the significantly higher proportion of female participants in this subgroup (89% vs. 59% in the negatives, Table 4, Fig. 3). Finally, patients with combined tissue positivity (defined as reactivity against any tissue type) exhibited higher C-reactive protein (CRP) levels (2.05 vs. 1.15 mg/l) and a greater frequency of anosmia and ageusia (75.5% vs. 37.5%) compared to those who were negative across all tissues (Table 1).

**Table 1:** Demographical and clinical characteristics of the long COVID cohorts. The data were grouped by overall autoreactivity status (Western blot, positive versus negative). Uncommon abbreviations: aab, autoantibody; PCFS, Post-COVID-19 Functional Status

	**Total**	**Western-blot aab. positive**	**Western-blot aab. negative**	***p*-value**
Age, years	45.5 (38.5–56)	47 (39.25–56)	41.5 (33–50)	0.183
Number of female patients (%)	42 (70%)	35 (70%)	7 (70%)	> 0.999
BMI, kg/m^2^	26.41 (23.8–29.75)	26.42 (24.02–30.23)	26.2 (22.23–27.75)	0.306
Other morbidities	0 (0–1)	0 (0–1)	1 (0–1)	0.961
Total COVID symptoms	6 (4–7)	6 (4–8)	6 (4–7)	0.588
Days hospitalized	0 (0–5)	0 (0–4)	0 (0–6.75)	0.415
WBC, G/L	6.4 (5.19–7.81)	6.6 (5.2–7.86)	5.94 (4.84–6.77)	0.329
Neutrophil, G/L	3.8 (2.91–5.11)	3.97 (2.95–5.2)	3.36 (2.5–3.98)	0.197
Lymphocyte, G/L	1.83 (1.48–2.28)	1.83 (1.47–2.19)	1.91 (1.61–2.54)	0.541
Monocyte, G/L	0.34 (0.28–0.41)	0.34 (0.28–0.41)	0.37 (0.29–0.41)	0.803
Eosinophil, G/L	0.12 (0.07–0.19)	0.13 (0.07–0.2)	0.08 (0.05–0.11)	0.097
NLR	137.5 (131–147.25)	137 (131–147.5)	138 (135–146)	0.762
Hemoglobin, g/L	1.5 (0.88–3.53)	2.05 (0.93–4.3)	1.15 (0.75–1.2)	**0.031**
CRP, mg/L	311 (223.75–440.5)	304.5 (221.25–457.75)	336.5 (247.5–377.25)	0.714
d-dimer, µg/L	2.04 (1.62–2.75)	2.13 (1.64–2.82)	1.86 (1.47–1.97)	0.080
Thrombin clotting time, s	246 (199–286.5)	246 (199–287.5)	246 (213–276.25)	0.761
Creatinine, µmol/L	65.5 (56.5–75.25)	66.5 (58–78.25)	63 (54–68.5)	0.240
ALT, U/L	23 (16–38)	20.5 (16–38.75)	36 (22–37)	0.528
AST, U/L	21 (16–28.25)	21.5 (16.25–27)	18.5 (16.25–29)	0.957
GGT, U/L	25 (14–48)	25 (14–47.75)	26 (14–47.25)	0.796
LDH, U/L	319.5 (287.75–367.5)	328.5 (287.25–362)	310.5 (300.5–367.25)	0.978
CK, U/L	74 (57.25–112.25)	75.5 (56.75–114)	72.5 (58.5–92.75)	0.907
BUN, mmol/L	4.18 (3.61–5.14)	4.18 (3.59–4.97)	4.58 (3.77–5.15)	0.553
Troponin T, ng/L	3.66 (3–4.69)	3.68 (3–4.67)	3.38 (3–5.71)	0.653
TSH, mU/L	1.93 (1.54–2.49)	1.97 (1.53–2.59)	1.88 (1.61–2.43)	0.872
ACE2, mU/L	38.76 (34.38–46.72)	38.76 (34.28–46.74)	39.92 (35.34–45.47)	0.774
Chalder total fatigue score	17 (13–20)	17.5 (14–21.5)	14 (11.5–16.5)	0.201
PCFS	2 (1–2)	2 (1–2)	2 (1–2)	0.699
Weakness/fatigue, *n* (%)	48 (84.2%)	43 (87.8%)	5 (62.5%)	0.103
Fever, *n* (%)	35 (61.4%)	31 (63.3%)	4 (50.0%)	0.698
Cough, *n* (%)	30 (52.6%)	25 (51.0%)	5 (62.5%)	0.709
Sore throat, *n* (%)	10 (17.5%)	8 (16.3%)	2 (25.0%)	0.619
Anosmia/Ageusia, *n* (%)	40 (70.2%)	37 (75.5%)	3 (37.5%)	**0.043**
Rhinorrhea, *n* (%)	9 (15.8%)	8 (16.3%)	1 (12.5%)	> 0.999
Shortness of breath, *n* (%)	18 (31.6%)	15 (30.6%)	3 (37.5%)	0.698
Chest pain, *n* (%)	13 (22.8%)	11 (22.4%)	2 (25.0%)	> 0.999
Myalgia, *n* (%)	33 (57.9%)	31 (63.3%)	2 (25.0%)	0.059
Arthralgia, *n* (%)	10 (17.5%)	8 (16.3%)	2 (25.0%)	0.619
Cutaneous pain, *n* (%)	16 (28.1%)	15 (30.6%)	1 (12.5%)	0.420
Diarrhea, *n* (%)	22 (38.6%)	17 (34.7%)	5 (62.5%)	0.239
Headache, *n* (%)	29 (50.9%)	25 (51.0%)	4 (50.0%)	> 0.999
Vomiting, *n* (%)	9 (15.8%)	6 (12.2%)	3 (37.5%)	0.103
Hypertension, *n* (%)	13 (27.7%)	13 (32.5%)	0 (0%)	0.166

**Table 2:** Demographical and clinical characteristics of the long COVID cohort analyzed by Western blot on cardiac tissues. The data were grouped by cardiac-autoreactivity status (positive versus negative). Uncommon abbreviations: aab, autoantibody; PCFS, Post-COVID-19 Functional Status

	**Total**	**Heart aab. positive**	**Heart aab. negative**	***p*-value**
Age, years	50 (42–57.25)	50 (43.5–58)	49 (42–56)	0.360
Number of female patients (%)	76 (66.7%)	44 (64.7%)	32 (69.6%)	0.687
BMI, kg/m^2^	26.4 (23.34–29.72)	27.61 (24.17–30.91)	25.56 (22.62–28.08)	**0.025**
Other morbidities	1 (0–1)	1 (0–2)	0 (0–1)	0.098
Total COVID symptoms	5.5 (4–7)	6 (3.25–8)	5 (4–7)	0.649
Days hospitalized	0 (0–4)	0 (0–4)	0 (0–5)	0.951
WBC, G/L	6.31 (5.08–7.76)	6.7 (5.06–8)	6.19 (5.17–7.01)	0.362
Neutrophil, G/L	3.72 (2.96–4.78)	3.76 (2.86–5.22)	3.72 (3.28–4.17)	0.482
Lymphocyte, G/L	1.85 (1.44–2.22)	1.85 (1.45–2.24)	1.9 (1.46–2.09)	0.946
Monocyte, G/L	0.33 (0.28–0.42)	0.35 (0.28–0.43)	0.32 (0.28–0.38)	0.320
Eosinophil, G/L	0.11 (0.07–0.19)	0.12 (0.07–0.23)	0.09 (0.06–0.18)	0.059
NLR	2.02 (1.64–2.71)	2.1 (1.62–2.82)	1.96 (1.66–2.37)	0.330
Hemoglobin, g/L	137 (131–146)	138 (131–147)	136 (130–143)	0.199
CRP, mg/L	1.5 (0.9–3.33)	1.9 (1–4.35)	1.2 (0.73–2.2)	**0.028**
d-dimer, µg/L	321.5 (225–495.5)	313 (226.5–493.5)	351 (224–486)	0.863
Thrombin clotting time, s	246 (214.75–288.5)	246 (212.75–289)	246 (219–281)	0.910
Creatinine, µmol/L	67 (56.25–77.25)	68 (57.75–79)	64 (55–76)	0.332
ALT, U/L	22 (16–37)	22.5 (18–38)	20 (13.5–36)	0.514
AST, U/L	21 (17–25)	21 (17–25)	20 (17–27)	0.857
GGT, U/L	22 (14–41.5)	24 (15–46.5)	18 (14–36)	0.194
LDH, U/L	333 (299–372.75)	332 (291.75–360)	338 (306.25–384)	0.292
CK, U/L	83 (60–127)	87 (61–127)	73 (58.5–124)	0.970
BUN, mmol/L	4.33 (3.7–5.32)	4.26 (3.67–5.36)	4.65 (3.74–5.2)	0.328
Troponin T, ng/L	3.91 (3–6.3)	3.86 (3–6.09)	3.91 (3.15–6.41)	0.577
TSH, mU/L	1.93 (1.54–2.42)	1.9 (1.43–2.43)	1.95 (1.62–2.4)	0.361
ACE2, mU/L	40.76 (35.26–48.29)	41.09 (35.6–50.91)	39.83 (35.14–47.39)	0.480
Chalder total fatigue score	17 (12.25–20)	17 (13–20.5)	15 (11.25–19)	0.120
PCFS	1 (1–2)	2 (1–2)	1 (1–2)	0.725
Weakness/fatigue, *n* (%)	81 (71.1%)	52 (76.5%)	29 (63%)	0.143
Fever, *n* (%)	65 (57%)	40 (58.8%)	25 (54.3%)	0.701
Cough, *n* (%)	58 (50.9%)	37 (54.4%)	21 (45.7%)	0.446
Sore throat, *n* (%)	20 (17.5%)	11 (16.2%)	9 (19.6%)	0.802
Anosmia/Ageusia, *n* (%)	70 (61.4%)	45 (66.2%)	25 (54.3%)	0.241
Rhinorrhea, *n* (%)	19 (16.7%)	12 (17.6%)	7 (15.2%)	0.802
Shortness of breath, *n* (%)	30 (26.3%)	16 (23.5%)	14 (30.4%)	0.516
Chest pain, *n* (%)	23 (20.2%)	12 (17.6%)	11 (23.9%)	0.479
Myalgia, *n* (%)	57 (50%)	36 (52.9%)	21 (45.7%)	0.567
Arthralgia, *n* (%)	21 (18.4%)	13 (19.1%)	8 (17.4%)	> 0.999
Cutaneous pain, *n* (%)	23 (20.2%)	15 (22.1%)	8 (17.4%)	0.638
Diarrhea, *n* (%)	35 (30.7%)	19 (27.9%)	16 (34.8%)	0.535
Headache, *n* (%)	44 (38.6%)	32 (47.1%)	12 (26.1%)	**0.031**
Vomiting, *n* (%)	18 (15.8%)	9 (13.2%)	9 (19.6%)	0.436
Hypertension, *n* (%)	35 (30.7%)	26 (38.2%)	9 (19.6%)	**0.042**

**Table 3:** Demographical and clinical characteristics of long COVID cohort analyzed by Western blot on vascular tissues (internal mammary arteries). The data were grouped by vascular-autoreactivity status (positive versus negative). Uncommon abbreviations: aab, autoantibody; PCFS, Post-COVID-19 Functional Status

	**Total**	**Artery aab. positive**	**Artery aab. negative**	***p*-value**
Age, years	46 (39–56)	46 (43–55)	47 (37.5–56.75)	0.638
Number of female patients (%)	49 (71.01%)	16 (69.57%)	33 (71.74%)	> 0.999
BMI, kg/m^2^	26.88 (24.02–29.76)	27.51 (24.34–31.40)	26.68 (24.04–29.74)	0.891
Other morbidities	0.5 (0–1)	0 (0–1)	1 (0–1)	0.956
Total COVID symptoms	6 (4–8)	6 (5–7.75)	6 (4–7.5)	0.395
Days hospitalized	23 (31.94%)	8 (33.33%)	15 (31.25%)	> 0.999
WBC, G/L	6.20 (5.06–7.78)	7.74 (5.13–8.34)	6.06 (5.02–7.46)	0.067
Neutrophil, G/L	3.79 (2.91–5.10)	4.84 (3.24–5.65)	3.56 (2.88–4.548)	0.109
Lymphocyte, G/L	1.85 (1.42–2.27)	1.81 (1.48–2.08)	1.85 (1.416–2.29)	0.927
Monocyte, G/L	0.34 (0.28–0.41)	0.37 (0.31–0.40)	0.33 (0.27–0.42)	0.301
Eosinophil, G/L	0.12 (0.07–0.19)	0.13 (0.09–0.21)	0.12 (0.06–0.19)	0.330
NLR	2.05 (1.64–2.74)	2.18 (1.75–3.06)	2.01 (1.55–2.54)	0.215
Hemoglobin, g/L	137 (131–147)	139 (133–150)	137 (131–146)	0.403
CRP, mg/L	1.5 (0.8–3.5)	1.9 (1.25–3.75)	1.35 (0.8–3.48)	0.320
d-dimer, µg/L	313 (230–454)	276 (208–346.5)	361 (233.5–459)	0.171
Thrombin clotting time, s	246 (207–281)	252 (227–270)	238.5 (199.5–284.75)	0.384
Creatinine, µmol/L	67 (55–77)	73 (59.5–79.5)	65.5 (55.25–75.75)	0.395
ALT, U/L	22 (16–38)	22 (13–52)	22 (17–37)	0.869
AST, U/L	21 (17–27)	23 (17.5–28)	21 (16–26.5)	0.387
GGT, U/L	23 (14–47)	25 (17–52)	22 (14–44.5)	0.324
LDH, U/L	326 (293–363)	338 (308–394.5)	321 (284.75–361)	0.590
CK, U/L	77 (60–122)	65 (56.5–104)	79.5 (60.75–127)	0.369
BUN, mmol/L	4.26 (3.68–5.17)	3.63 (3.31–4.16)	4.52 (3.8–5.19)	**0.012**
Troponin T, ng/L	3.67 (3–5.37)	3.88 (3.56–5.04)	3.59 (3–5.7)	0.303
TSH, mU/L	1.89 (1.52–2.44)	1.89 (1.55–2.27)	1.91 (1.52–2.9)	0.330
ACE2, mU/L	40.14 (33.66–46.81)	46.26 (35.39–55.56)	38.47 (32.32–43.7)	0.503
Chalder total fatigue score	17 (13–20)	16 (13.5–18.5)	17 (13.25–20.75)	0.636
PCFS	2 (1–2)	2 (1–2)	2 (1–2)	0.525
Weakness/fatigue, *n* (%)	23 (33.33%)	19 (33.33%)	4 (33.33%)	> 0.999
Fever, *n* (%)	42 (60.87%)	15 (65.22%)	27 (58.7%)	0.794
Cough, *n* (%)	37 (53.62%)	13 (56.52%)	24 (52.17%)	0.801
Sore throat, *n* (%)	14 (20.29%)	6 (26.09%)	8 (17.39%)	0.527
Anosmia/Ageusia, *n* (%)	46 (66.67%)	17 (73.91%)	29 (63.04%)	0.426
Rhinorrhea, *n* (%)	13 (18.84%)	4 (17.39%)	9 (19.57%)	> 0.999
Shortness of breath, *n* (%)	22 (31.88%)	4 (17.39%)	18 (39.13%)	0.100
Chest pain, *n* (%)	17 (24.64%)	4 (17.39%)	13 (28.26%)	0.387
Myalgia, *n* (%)	41 (59.42%)	13 (56.52%)	28 (60.87%)	0.798
Arthralgia, *n* (%)	12 (17.39%)	7 (30.43%)	5 (10.87%)	0.088
Cutaneous pain, *n* (%)	19 (27.54%)	7 (30.43%)	12 (26.09%)	0.778
Diarrhea, *n* (%)	24 (34.78%)	10 (43.48%)	14 (30.43%)	0.298
Headache, *n* (%)	36 (52.17%)	12 (52.17%)	24 (52.17%)	> 0.999
Vomiting, *n* (%)	12 (17.39%)	2 (8.7%)	10 (21.74%)	0.312
Hypertension, *n* (%)	17 (26.98%)	7 (33.33%)	10 (23.81%)	0.549

**Table 4:** Demographical and clinical characteristics of long COVID cohort analyzed by Western blot on pulmonary tissues. The data were grouped by pulmonary-autoreactivity status (positive versus negative). Uncommon abbreviations: aab, autoantibody; PCFS, Post-COVID-19 Functional Status

	**Total**	**Lung aab. positive**	**Lung aab. negative**	***p*-value**
Age, years	49 (42–57.75)	49 (43.75–58.25)	51 (42–56.75)	0.894
Number of female patients (%)	57 (68.7%)	24 (88.9%)	33 (58.9%)	**0.006**
BMI, kg/m^2^	26.38 (23.36–29.75)	26.47 (22.87–29.7)	26.35 (24.02–30.32)	0.967
Other morbidities	1 (0–2)	0 (0–1)	1 (0–2)	0.541
Total COVID symptoms	6 (4–7)	5 (4–8)	6 (4–7)	0.479
Days hospitalized	0 (0–5)	0 (0–2)	0 (0–5)	0.223
WBC, G/L	6.34 (5.1–7.72)	6.12 (5.08–7.5)	6.45 (5.1–7.77)	0.550
Neutrophil, G/L	3.78 (2.95–4.96)	3.81 (2.87–4.54)	3.74 (3.19–5.14)	0.403
Lymphocyte, G/L	1.79 (1.43–2.14)	1.93 (1.64–2.17)	1.72 (1.38–2.1)	0.133
Monocyte, G/L	0.34 (0.28–0.42)	0.32 (0.28–0.4)	0.35 (0.29–0.43)	0.233
Eosinophil, G/L	0.12 (0.07–0.2)	0.14 (0.07–0.19)	0.11 (0.08–0.23)	0.986
NLR	2.09 (1.65–2.78)	1.97 (1.56–2.41)	2.14 (1.67–2.96)	0.121
Hemoglobin, g/L	137 (131–144.75)	134.5 (128.25–140)	138 (132–145.75)	0.083
CRP, mg/L	1.7 (0.9–3.5)	1.7 (0.7–3.25)	1.7 (1–3.6)	0.500
d-dimer, µg/L	342 (228–486)	312 (205–433.5)	355 (239.25–517.75)	0.224
Thrombin clotting time, s	246.5 (209.75–295.25)	254.5 (233.75–307.75)	244 (205–286.25)	0.206
Creatinine, µmol/L	66 (55–77)	63 (55–69.25)	68.5 (57.25–80.5)	**0.030**
ALT, U/L	24 (16.25–37)	19.5 (16–26.5)	27.5 (17.25–38)	0.183
AST, U/L	21 (17–27)	20.5 (16–23.25)	21.5 (17–29)	0.170
GGT, U/L	24.5 (14–45.75)	24 (12.75–30.75)	24.5 (14.25–48)	0.103
LDH, U/L	334.5 (297.5–383.75)	324 (282.25–344.5)	337 (305.25–390.5)	0.103
CK, U/L	77 (59–120.5)	71 (59–104)	88 (59.5–130.25)	0.196
BUN, mmol/L	4.3 (3.66–5.2)	4.37 (3.58–5.19)	4.3 (3.7–5.36)	0.654
Troponin T, ng/L	3.92 (3–6.09)	3.55 (3–4.59)	4.03 (3.25–6.72)	**0.033**
TSH, mU/L	2.03 (1.55–2.44)	2.1 (1.65–3.44)	1.95 (1.55–2.34)	0.159
ACE2, mU/L	40.54 (35.3–47.39)	37.7 (34.03–43.64)	41.63 (37.33–52.8)	0.068
Chalder total fatigue score	17 (13–20)	17.5 (14.5–21.25)	16 (12–19)	0.076
PCFS	2 (1–2)	2 (1–2)	1.5 (1–2)	0.216
Weakness/fatigue, *n* (%)	66 (79.5%)	22 (81.5%)	44 (78.6%)	> 0.999
Fever, *n* (%)	54 (65.1%)	18 (66.7%)	36 (64.3%)	> 0.999
Cough, *n* (%)	49 (59.0%)	16 (59.3%)	33 (58.9%)	> 0.999
Sore throat, *n* (%)	14 (16.9%)	8 (29.6%)	6 (10.7%)	0.057
Anosmia/Ageusia, *n* (%)	58 (69.9%)	20 (74.1%)	38 (67.9%)	0.619
Rhinorrhea, *n* (%)	15 (18.1%)	6 (22.2%)	9 (16.1%)	0.549
Shortness of breath, *n* (%)	28 (33.7%)	8 (29.6%)	20 (35.7%)	0.629
Chest pain, *n* (%)	17 (20.5%)	8 (29.6%)	9 (16.1%)	0.161
Myalgia, *n* (%)	42 (50.6%)	17 (63.0%)	25 (44.6%)	0.160
Arthralgia, *n* (%)	16 (19.3%)	5 (18.5%)	11 (19.6%)	> 0.999
Cutaneous pain, *n* (%)	19 (22.9%)	8 (29.6%)	11 (19.6%)	0.404
Diarrhea, *n* (%)	31 (37.3%)	8 (29.6%)	23 (41.1%)	0.344
Headache, *n* (%)	38 (45.8%)	12 (44.4%)	26 (46.4%)	> 0.999
Vomiting, *n* (%)	16 (19.3%)	2 (7.4%)	14 (25.0%)	0.076
Hypertension, *n* (%)	29 (36.7%)	9 (34.6%)	20 (37.7%)	> 0.999

**Fig. 4:**
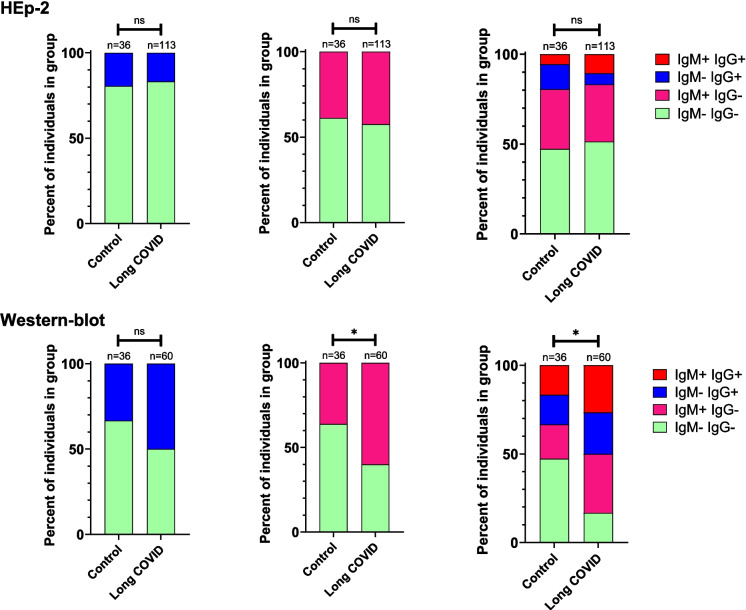
Comparative analysis of tissue-specific Western blotting and standard HEp-2 cell indirect immunofluorescence. Stacked bar charts represent the percentage of patients categorized by autoantibody profile in control (*n* = 36) and long COVID (*n* = 60–113) cohorts. The panels contrast the detection frequency using the specialized tissue-antigen Western blot method against results from standard clinical HEp-2 cell assays. The color scheme indicates serological status, where *green* represents samples negative for both IgM and IgG, *magenta* denotes IgM-only positivity, *blue* represents IgG-only positivity, and *red* shows double-positivity for both IgM and IgG. Statistical significance between groups was determined using the chi-square test, with “ns” indicating no significant difference between the cohorts

The Western blot method was also compared to a more routine autoantibody detection technique, the ANA HEp-2 assay. This assay did not differentiate between patients with or without autoantibodies (regardless of the tissue), which was in contrast with the Western blot–based method. In particular, the occurrence of both IgG and IgM autoantibodies was similar in the long COVID and control populations, and there was no difference in the overall immunological profile when both isotypes were considered (Fig. 4). On the contrary, while Western-blot assay demonstrated statistically similar occurrence of IgG autoantibodies (albeit numerically higher in the long COVID patients, 50% versus 33%, *p* = 0.14 Fig. 4), the occurrence of IgM autoantibodies was significantly higher (60% versus 36%, Fig. 4, *p* < 0.05). More importantly, the overall prevalence of autoantibodies were 83% in the long COVID patients, while 53% in the controls (*p* < 0.05, Fig. 4).

**Fig. 5:**
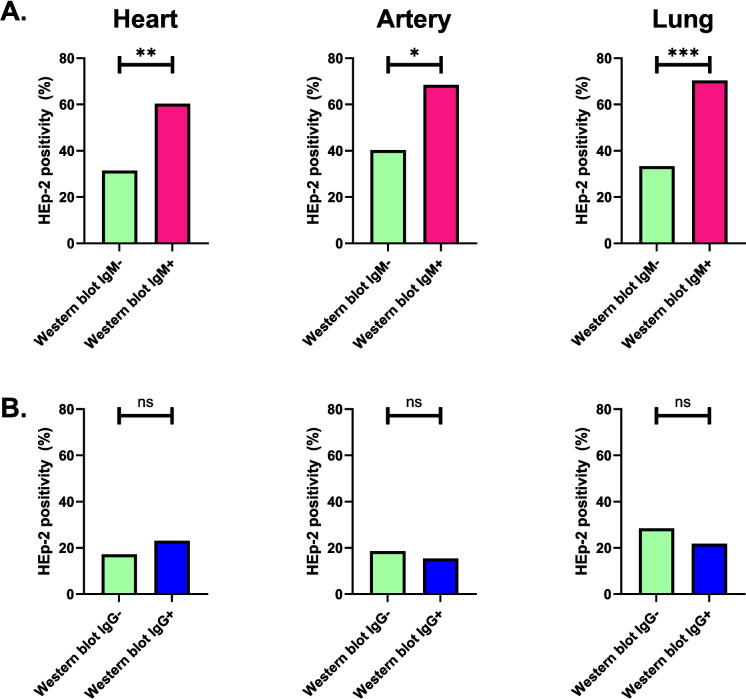
Analysis of the relationship between tissue-specific Western-blot autoantibody status and HEp-2 cell positivity. **A** The upper bar charts illustrate the percentage of HEp-2 positivity in patients stratified by their IgM status for heart, artery, and lung tissues. **B** The lower charts contrast HEp-2 positivity rates relative to tissue-specific IgG status. *Green bars* represent the percentage of HEp-2 positive patients in the IgM-negative (IgM −) or IgG-negative groups, while *magenta bars* represent the percentage in the IgM-positive (IgM +) or IgG-positive groups. Statistical significance between the groups was assessed using the chi-square test, where asterisks indicate significant differences (*p < 0.05, **p < 0.01, ***p < 0.001) and “ns” indicates a non-significant result

Next, we assessed the concordance between the two diagnostic methods. A significant correlation was found regarding IgM positivity: individuals testing positive for IgM autoantibodies by Western blot were more likely to show IgM positivity in the ANA HEp-2 assay (Fig. 5A). In contrast, no such concordance was observed for IgG autoantibodies (Fig. 5B). Regarding clinical associations, ANA HEp-2 positivity showed no significant correlation with specific clinical symptoms. However, demographic and inflammatory markers differed: age (53 versus 46 years), the proportion of females (77% versus 57%), and CRP levels (2.0 versus 1.2 mg/L) were higher in the ANA-positive Long COVID patients (Table 5).

**Table 5:** Demographical and clinical characteristics of long COVID cohort analyzed by ANA HEp-2 assay. The data were grouped by HEp-2 reactivity (positive versus negative). Uncommon abbreviations: ANA HEp2, Antinuclear-antibody patterns on human epithelial type-2 cells; PCFS, Post-COVID-19 Functional Status

	**Total**	**HEp-2 positive**	**HEp-2 negative**	***p*-value**
Age, years	49 (42–57)	53 (44.5–59.5)	46 (37.5–54)	**0.015**
Number of female patients (%)	67 (66.3%)	37 (77.1%)	30 (56.6%)	**0.036**
BMI, kg/m^2^	26.45 (23.37–29.76)	27.44 (24.49–30.09)	26.12 (23.04–29.68)	0.292
Other morbidities	1 (0–1)	1 (0–1)	0.5 (0–1.25)	0.996
Total COVID symptoms	5 (4–7)	5 (3–8)	6 (4–7)	0.931
Days hospitalized	0 (0–4)	0 (0–4)	0 (0–4)	0.628
WBC, G/L	6.31 (5.09–7.67)	6.38 (5.15–7.51)	6.3 (5.08–7.77)	0.900
Neutrophil, G/L	3.79 (2.92–4.84)	3.93 (2.92–4.82)	3.56 (3.1–5.02)	0.690
Lymphocyte, G/L	1.86 (1.45–2.27)	1.89 (1.5–2.12)	1.83 (1.39–2.39)	0.940
Monocyte, G/L	0.34 (0.28–0.42)	0.33 (0.28–0.43)	0.34 (0.28–0.4)	0.919
Eosinophil, G/L	0.12 (0.07–0.19)	0.12 (0.07–0.19)	0.12 (0.07–0.2)	0.709
NLR	2.02 (1.64–2.74)	2.05 (1.66–2.74)	1.99 (1.56–2.66)	0.887
Hemoglobin, g/L	137 (131–146)	136 (130.5–147.5)	138 (131.25–144.75)	0.715
CRP, mg/L	1.6 (0.9–3.43)	1.95 (1.23–4.3)	1.2 (0.7–3.2)	**0.038**
d-dimer, µg/L	329.5 (224–490.75)	344 (229.75–517.5)	305 (220.25–453.25)	0.513
Thrombin clotting time, s	246 (214–293)	252 (220–300.5)	235.5 (208.25–281)	0.180
Creatinine, µmol/L	67 (56–77)	70 (57.5–77)	65 (55–76.75)	0.520
ALT, U/L	23 (16–37)	21 (17–33.5)	24 (15.25–37.75)	0.820
AST, U/L	21 (17–25)	21 (16.5–25.5)	21 (17–25)	0.953
GGT, U/L	22 (14–43)	23 (15–52.5)	20.5 (14–35.5)	0.259
LDH, U/L	334 (299–383)	336 (307.5–384)	326 (283.25–363)	0.424
CK, U/L	86.5 (60.25–127)	82.5 (64–122.5)	90.5 (57.5–132.25)	0.691
BUN, mmol/L	4.47 (3.74–5.38)	4.3 (3.75–5.49)	4.6 (3.71–5.22)	0.978
Troponin T, ng/L	3.91 (3–6.21)	3.67 (3–5.99)	3.93 (3.2–6.5)	0.360
TSH, mU/L	1.93 (1.54–2.41)	1.71 (1.37–2.35)	2.06 (1.65–2.44)	0.073
ACE2, mU/L	40.81 (35.26–50.34)	40.81 (32.07–51.75)	40.87 (36.78–47.12)	0.573
Chalder total fatigue score	17 (13–20)	17 (13–20)	17 (13–20)	0.537
PCFS	2 (1–2)	1 (1–2)	2 (1–2)	0.178
Weakness/fatigue, *n* (%)	80 (79.2%)	39 (81.3%)	41 (77.4%)	0.807
Fever, *n* (%)	65 (64.4%)	27 (56.3%)	38 (71.7%)	0.145
Cough, *n* (%)	56 (55.4%)	27 (56.3%)	29 (54.7%)	> 0.999
Sore throat, *n* (%)	20 (19.8%)	12 (25.0%)	8 (15.1%)	0.225
Anosmia/Ageusia, *n* (%)	68 (67.3%)	31 (64.6%)	37 (69.8%)	0.672
Rhinorrhea, *n* (%)	18 (17.8%)	9 (18.8%)	9 (17.0%)	> 0.999
Shortness of breath, *n* (%)	29 (28.7%)	13 (27.1%)	16 (30.2%)	0.827
Chest pain, *n* (%)	23 (22.8%)	13 (27.1%)	10 (18.9%)	0.352
Myalgia, *n* (%)	56 (55.4%)	27 (56.3%)	29 (54.7%)	> 0.999
Arthralgia, *n* (%)	21 (20.8%)	13 (27.1%)	8 (15.1%)	0.151
Cutaneous pain, *n* (%)	23 (22.8%)	8 (16.7%)	15 (28.3%)	0.235
Diarrhea, *n* (%)	35 (34.7%)	15 (31.3%)	20 (37.7%)	0.535
Headache, *n* (%)	44 (43.6%)	23 (47.9%)	21 (39.6%)	0.428
Vomiting, *n* (%)	18 (17.8%)	6 (12.5%)	12 (22.6%)	0.205
Hypertension, *n* (%)	34 (34.7%)	16 (34.0%)	18 (35.3%)	> 0.999

**Fig. 6:**
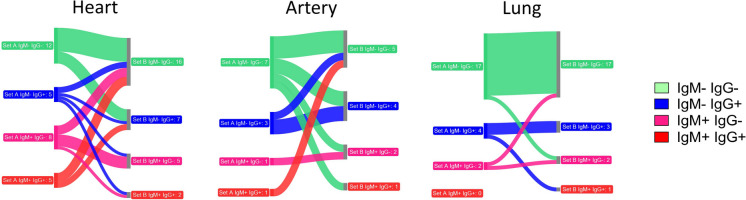
Sankey diagrams illustrating the longitudinal changes in tissue-specific autoantibody profiles. The diagrams track the progression of IgM and IgG reactivity in heart, artery, and lung tissues between two visits, labeled as set A and set B (mean follow-up period of 141 days). The width of the connecting flows represents the number of patients maintaining or shifting their serological status between visits. Color coding denotes specific antibody combinations: *green* shows double-negative status (IgM − IgG −), *blue* indicates IgG-only positivity (IgM − IgG +), *magenta* represents IgM-only positivity (IgM + IgG −), and *red* indicates positivity for both IgM and IgG (IgM + IgG +)

Finally, follow-up serum samples were obtained from a subgroup of patients approximately 141 days after the baseline visit (*n* = 30 for cardiac tissue, *n* = 12 for vascular tissue, *n* = 23 for lung tissue), which provided an insight into the temporal dynamics of autoimmunity. The expected immunological behavior (maintenance of seronegativity, clearance of autoantibodies, and IgM-to-IgG class switching) was most evident in the case of lung-specific autoantibodies. However, the pattern was markedly different for cardiac antigens, which showed persistent IgM reactivity and the frequent emergence of new autoantibodies. The vascular tissue exhibited an intermediate profile (Fig. 6).

## Discussion

The present study identifies several important findings regarding the immunological landscape of long COVID. First, a substantial proportion of patients exhibited tissue-specific autoantibodies with a marked predilection for the cardiovascular system. Second, this autoreactivity was frequently polyreactive — defined as the simultaneous targeting of multiple tissue antigens — a pattern that was particularly pronounced against cardiac proteins. Third, the majority of detected autoantibodies were of the IgM isotype, suggesting persistent antigenic stimulation, failed or incomplete immunoglobulin class switching, or an atypical extrafollicular humoral response. Fourth, routine ANA HEp-2 testing failed to detect this autoreactivity, highlighting a significant diagnostic gap in standard serological screening for long COVID-associated immune dysregulation. Fifth, clinically relevant associations were observed between tissue-specific autoreactivity and neurovascular manifestations, including hypertension, headache, and persistent anosmia and ageusia, suggesting a potential link between humoral autoimmunity and end-organ neurovascular dysfunction. Sixth, standard inflammatory markers such as CRP were only marginally elevated in seropositive individuals, indicating that this autoreactive immune signature may remain entirely undetected by conventional inflammatory screening, further underscoring the need for more targeted diagnostic approaches.

While the acute phase of SARS-CoV-2 infection has been shown to trigger a broad spectrum of tissue-specific autoimmune responses — including anti-cardiac [9] and anti-pulmonary autoantibodies [10] — data on structural tissue-specific autoreactivity in long COVID remain scarce. Research has predominantly focused on systemic and non-organ-specific markers, including antinuclear antibodies, autoantibodies targeting cytokines such as anti-interferon-α−2 [31–36], and functional autoantibodies directed against G-protein-coupled receptors, including adrenergic and muscarinic receptors, the latter suggesting a pathogenic mechanism involving vascular and autonomic dysregulation [37]. Structural autoantigens derived from specific organ systems have received comparatively little attention in this context. Our study addresses this gap by simultaneously screening for autoantibodies targeting cardiac, pulmonary, and vascular tissue homogenates within the same patient cohort — an approach that, to our knowledge, has not previously been applied in long COVID research; thus, it captures a dimension of tissue-directed autoreactivity inconspicuous to conventional serological screening tools.

Tissue-specific autoantibodies were detected in the vast majority of patients, with cardiac autoreactivity representing the most frequent response — observed in over half of the cohort — followed by pulmonary autoreactivity in a substantial subset and vascular autoreactivity in over a third of patients, the latter being significantly more prevalent compared to controls. Low-level autoantibody reactivity was also detectable in some control samples, consistent with physiological autoreactivity in healthy individuals [38], potentially further influenced by prior occupational immune exposures [39] and by the exposure of linear epitopes under denaturing conditions inherent to Western blotting [40]. The autoreactive profile in long COVID patients was, however, characterized by markedly greater breadth, tissue specificity, and distinct clinical associations, supporting the interpretation that SARS-CoV-2 infection may promote sustained tissue-directed immune responses through tissue injury-induced antigen exposure and subsequent epitope spreading [41].

A fundamental challenge in interpreting tissue-based Western blot studies of this kind is distinguishing physiological from potentially pathological autoreactivity, and determining whether the observed signatures represent a long-COVID-specific immunological phenomenon or a broader post-viral immune response. Physiological autoreactivity serves homeostatic functions and is present at low levels in healthy individuals, while pathological autoreactivity is generally characterized by greater antigen specificity, isotype diversity, persistence beyond the expected window of post-infectious immune resolution, and association with clinical manifestations. Several features of the autoreactive profile observed in our cohort argue against a purely physiological interpretation — including the significantly greater prevalence and breadth compared to pre-pandemic controls, heterogeneous isotype dynamics, and statistically significant associations with defined neurovascular clinical phenotypes. Post-infectious autoantibody induction is furthermore not unique to SARS-CoV-2 — EBV, influenza, and arboviral infections including chikungunya and Zika have all been associated with tissue-directed autoreactive responses and cardiovascular complications extending beyond the acute phase [42–46]. It remains plausible that long COVID represents the symptomatic extreme of a post-infectious autoreactive continuum, in which the magnitude, persistence, and tissue specificity of the humoral response — rather than its mere presence — distinguish those who develop chronic sequelae from those who recover. Critically, while autoreactive responses following common respiratory infections typically resolve within weeks, the persistence of tissue-specific autoantibodies and associated symptoms beyond 30 days has been shown to discriminate long COVID from uncomplicated convalescence [47], suggesting that the failure of normal immune contraction — rather than initial induction — may be the defining immunological feature of long COVID. Definitive attribution to long-COVID-specific pathophysiology must nonetheless await prospective studies incorporating parallel sampling of infection-matched recovered and long COVID cohorts.

The functional and clinical significance of the detected autoantibodies remains an equally important interpretive question. At present, it is not possible to determine from our data alone whether these autoantibodies are pathogenic drivers of tissue injury, epiphenomenal markers of ongoing immune activation and tissue damage, or components of a physiological damage-clearance response that has become dysregulated. Their association with specific neurovascular clinical manifestations — including hypertension, headache, and persistent anosmia and ageusia — suggests that at least a subset may carry pathophysiological relevance rather than representing purely incidental findings. However, Western blot-based detection of antibodies against tissue homogenates does not establish target antigen identity, functional activity, or tissue accessibility in vivo. Future studies employing antigen identification strategies such as immunoprecipitation-mass spectrometry or high-density antigen arrays, combined with functional assays such as endothelial activation or vascular permeability models, will be essential to determine whether these autoantibodies actively contribute to the microvascular and neurovascular dysfunction characteristic of long COVID, or whether they primarily serve as biomarkers of disease persistence and immune dysregulation.

The dominance of IgM autoantibodies represents one of the most distinctive immunological features of this study. Two competing mechanisms may explain this signature: the persistence of physiological natural antibodies involved in damage-associated molecular pattern (DAMP) clearance [48], or a pathological failure of class-switching recombination [31]. Our longitudinal data support the dysregulation hypothesis. While lung autoreactivity followed a predictable physiological course — stability of IgG with waning of IgM — cardiac and vascular antibody dynamics were markedly heterogeneous. The erratic behavior observed in these tissues, characterized by prolonged IgM persistence and the sudden de novo appearance of new isotypes, indicates a deviation from standard immune maturation and is in keeping with ongoing immunological dysregulation, although the precise mechanisms remain to be determined. A third mechanistic contributor worth considering is the extrafollicular B cell response pathway. In conditions of chronic immune activation and persistent antigen exposure, extrafollicular B cell pathways generate predominantly IgM-class antibodies with limited somatic hypermutation, bypassing the germinal center maturation required for effective class-switch recombination [49]. This mechanism has been implicated in pathological autoantibody production in systemic autoimmune diseases such as SLE, and may be similarly operative in the context of sustained post-infectious immune activation in long COVID. Persistent IgM autoreactivity has been documented across other post-infectious syndromes as well — in ME/CFS, an IgM-dominant immune response directed against oxidative stress-related neoepitopes has been interpreted as a marker of chronic immune activation [50], while in post-treatment Lyme disease syndrome, autoantibody responses targeting vascular and extracellular matrix antigens have been attributed to ongoing antigen presentation by damaged endothelial cells and synovial fibroblasts [51]. These parallels suggest that persistent IgM autoreactivity may represent a shared immunological feature of post-infectious states characterized by sustained tissue injury and inadequate immune resolution, rather than a phenomenon unique to long COVID.

Whether the detected IgM responses reflect true antigen-specific immune activation or, at least in part, the binding of natural low-affinity polyreactive antibodies to linear neoepitopes exposed under the denaturing conditions of SDS-PAGE warrants careful consideration. Natural IgM antibodies are inherently polyreactive and capable of binding a broad range of denatured protein epitopes, which may contribute to background signal in Western blot-based assays. Supplementary Fig. 2 directly addresses this concern: at low serum dilutions, a broad pattern of IgM and IgG reactivity is evident, reflecting the presence of low-affinity natural antibodies directed against a wide range of tissue epitopes. This nonspecific background reactivity is progressively eliminated with increasing dilution, becoming largely undetectable at approximately 1:300 — such that at the stringent 1:1000 dilution employed in our study, only a limited number of high-affinity interactions persist. This dilution-dependent pattern provides empirical evidence that the reported autoantibody signals represent affinity-matured, antigen-specific responses rather than low-affinity polyreactive binding. The observed tissue specificity, clinical associations, and longitudinal persistence of the IgM responses — features not typically associated with nonspecific natural antibody reactivity — further argue in favor of genuine antigen-specific immune engagement. Definitive distinction between physiological polyreactivity and true antigen-specific autoreactivity will nonetheless require higher-resolution methodologies, including affinity measurements, competitive inhibition assays, and epitope mapping.

The IgM-dominant pattern may additionally be understood in the context of age-related immune remodeling. Aging is associated with expansion of distinct B cell subsets, including age-associated B cells [52], as well as shifts toward extrafollicular and innate-like humoral responses. In parallel, the natural antibody repertoire undergoes qualitative and quantitative drift with age, characterized by increased baseline autoreactivity and altered antigen recognition breadth [53]. Within this framework, the autoreactivity observed in our cohort may reflect a convergence of post-infectious immune activation and age-dependent changes in humoral immune architecture, potentially lowering the threshold for sustained autoreactive responses following tissue injury [54].

The autoantibodies identified in this study are importantly distinct from those targeted by routine clinical serological assays. Standard ANA testing detects ubiquitous nuclear antigens using native conformational epitopes presented on intact cells, as in the HEp-2 substrate [55], whereas our Western blot approach identifies autoantibodies targeting denatured linear epitopes derived from cardiac, vascular, and pulmonary tissue homogenates. Given that these homogenates encompass a broad representation of the target tissue proteome, our approach functions as an unbiased screening platform for structural tissue autoantigens rather than a targeted assay for predefined specificities. The autoantibodies identified here, therefore, likely represent a distinct serological entity — rather than serving as markers of classic systemic autoimmune disorders such as SLE, they are better characterized as indicators of an organ-specific, post-infectious autoimmune process driven by SARS-CoV-2-associated tissue injury and antigen exposure.

The distribution of target antigens across this heterogeneous autoreactive profile, assessed by approximate molecular weight, was markedly divergent. IgG and IgM autoantibodies appeared to target antigens at distinct molecular weights across cardiac, vascular, and pulmonary tissues, with minimal overlap between isotypes or tissue types. This broad and diffuse pattern is difficult to reconcile with molecular mimicry as the primary driver of autoreactivity in our cohort [39]. Molecular mimicry, in its classical form, involves cross-reactive antibodies generated against viral epitopes sharing structural homology with specific host proteins — a mechanism that would be expected to produce autoreactivity concentrated at defined molecular weights corresponding to the mimicked host antigens, rather than the tissue-spanning pattern observed here. While molecular mimicry cannot be entirely excluded based on molecular weight distribution alone — as structurally homologous epitopes could theoretically occur across multiple proteins — the breadth and heterogeneity of the observed reactivity is more readily explained by mechanisms such as widespread tissue injury, bystander B cell activation, or epitope spreading following SARS-CoV-2-induced tissue damage, all of which would be expected to generate a broader and less antigen-specific autoreactive response.

Comparative analysis of the HEp-2 clinical assay and our Western blot approach revealed a clear divergence in diagnostic performance in the long COVID context. The HEp-2 assay showed no statistically significant differences between long COVID patients and controls across any isotype tested — IgG, IgM, or combined — with comparable positivity rates in both cohorts, indicating that this standard serological tool lacks the sensitivity and specificity required to detect or characterize the tissue-directed immune alterations observed in long COVID [56]. Patients testing positive for anti-tissue IgM by Western blot were, however, significantly more likely to exhibit HEp-2 reactivity compared to their IgM-negative counterparts. This parallel positivity should be interpreted with caution — given that HEp-2 positivity was equally prevalent in the control group, this co-reactivity most plausibly reflects a shared background of nonspecific polyclonal immune activation rather than a true overlap in antigen specificity, in keeping with the broad, low-affinity polyreactive IgM responses that characterize both physiological autoreactivity and early post-infectious immune activation. By focusing detection on structurally defined tissue antigens, the Western blot approach appears to overcome this limitation. To our knowledge, this represents the first direct comparative analysis of these two methodologies in the long COVID setting, and its most direct clinical implication is that a negative or nonspecific HEp-2 result does not exclude clinically relevant tissue-directed autoreactivity in this patient cohort.

Turning to clinical associations, cardiac autoreactivity was significantly linked to neurovascular manifestations — specifically headache and hypertension — both of which were more prevalent in the autoantibody-positive subgroup compared to seronegative patients. These findings align with emerging evidence identifying new-onset hypertension as an increasingly recognized post-COVID complication [57], and support a potential link between persistent humoral immune activation and vascular dysfunction. Classical cardiovascular symptoms such as palpitations, chest pain, and dyspnea did not show consistent associations with cardiac autoantibody status, suggesting that tissue-directed autoreactivity may preferentially engage vascular and neurovascular pathways rather than causing overt myocardial injury — a pattern more consistent with immune-mediated endothelial dysfunction than with direct cardiomyocyte targeting [58]. It should be acknowledged, however, that the heterogeneity of long COVID symptomatology and the exploratory nature of multiple symptom comparisons within a single cohort introduce a risk of false-positive associations. These clinical correlations should therefore be regarded as hypothesis-generating findings requiring replication in larger, prospectively designed studies with appropriate correction for multiple comparisons.

From a pathophysiological perspective, these findings are in keeping with an emerging microvascular model of long COVID [58], in which immune dysregulation, endothelial injury, and microvascular dysfunction form a self-reinforcing pathogenic cascade. In human pathological studies, long COVID has been associated with demonstrable microvascular injury across multiple organ systems — including capillary rarefaction documented by sublingual video microscopy up to 18 months after infection, reduced flow-mediated vasodilation, impaired endothelial progenitor cell function, and evidence of persistent microthrombotic and endotheliopathic processes [59]. At the cerebral level, persistent endothelial dysfunction has been specifically linked to impaired neurovascular coupling, blood–brain barrier disruption, and compromised cerebral perfusion, providing a documented human pathological substrate for the neurocognitive and neurovascular symptoms characteristic of long COVID [60]. Within this framework, tissue-directed autoreactivity — particularly if targeting endothelial or perivascular antigens — could contribute to the perpetuation of this endotheliopathic state by sustaining vascular inflammation, impairing endothelial repair mechanisms, and amplifying microvascular dysfunction beyond the initial phase of viral injury. Our data demonstrate an association between tissue-specific autoreactivity and neurovascular clinical manifestations, but do not establish causality — the precise contribution of the detected autoantibodies to the observed vascular pathology cannot be determined from the present study design alone.

The cardiac autoreactivity signal warrants particular interpretive attention in this context. Cardiac tissue homogenates contain a substantial microvascular component — including capillary endothelial cells, pericytes, and small arteriolar structures — that is inevitably co-homogenized with cardiomyocytes and interstitial tissue under standard preparation protocols. The detected autoantibodies may therefore, at least in part, reflect immune targeting of microvascular rather than myocardial antigens. The absence of associations with classical myocardial symptoms such as palpitations or chest pain, combined with growing evidence that microvascular injury represents the predominant cardiac pathological manifestation in the long COVID context [58], lends support to this interpretation — though it cannot be confirmed without antigen-specific identification studies. The internal mammary artery homogenate used as our vascular substrate represents large conduit vessel tissue differing substantially in antigenic composition from the microvasculature, meaning that microvascular-specific autoreactivity may not be fully captured by this preparation either. Future investigations employing microvascular-enriched tissue substrates — such as primary human coronary microvascular endothelial cell preparations — or immunohistochemical analysis of tissue sections will be needed to confirm whether the detected autoreactivity is genuinely directed against the microvascular compartment.

The present findings extend the limited existing evidence on tissue-directed autoreactivity in long COVID. While Wallukat et al. demonstrated the presence of functional autoantibodies in symptomatic patients compared to asymptomatic long COVID patients [37], their analysis did not examine associations with specific clinical manifestations — a gap the present study begins to address. The present study nonetheless identifies significant associations between cardiac autoreactivity and neurovascular manifestations, including hypertension and headache, and between combined tissue positivity and persistent anosmia and ageusia — findings that, while requiring replication, suggest that tissue-directed autoreactivity carries at least partial clinical relevance in this context. The absence of clear associations between pulmonary and vascular autoantibodies and clinical presentation likely reflects differences in antigen composition, the substantially different antigenic profile of large conduit vessel tissue compared to the microvasculature, or the inherently multifactorial nature of long COVID symptomatology. The limited number of significant clinical correlations overall is not unexpected and aligns with the broader literature, in which establishing direct links between specific autoantibodies and clinical symptoms has proven consistently challenging. Comprehensive studies utilizing high-throughput antigen profiling approaches such as REAP and phage immunoprecipitation have generally reported no significant differences in autoreactivity between long COVID patients and recovered individuals [61, 62], underscoring the difficulty of identifying disease-specific autoantibody signatures. The mere presence of autoantibodies does not necessarily precipitate a clinically manifest immune response — target antigen accessibility, antibody affinity, local inflammatory context, and the biological significance of the antigenic target all determine pathophysiological impact. While some reports have implicated traditional markers such as ANAs in predicting long COVID sequelae [63], their diagnostic utility remains controversial given their low specificity and variable prevalence in the general population [64, 65]. The pathogenesis of long COVID almost certainly involves mechanisms extending well beyond autoimmunity — including viral persistence, mitochondrial dysfunction, autonomic dysregulation, and microbiome alterations — and the autoantibody signatures identified here should be interpreted as one component of a complex and multifactorial pathological process. Functional autoantibodies against GPCRs represent a distinct and complementary pathophysiological mechanism that may operate in parallel with the structural tissue autoreactivity described here [66–70].

Comparative analysis of clinical laboratory parameters between autoantibody-positive and autoantibody-negative subgroups revealed several distinct patterns. Patients with anti-cardiac autoantibodies exhibited significantly higher BMI and CRP levels compared to their seronegative counterparts. Although the CRP difference reached statistical significance, values remained within the normal physiological range in both groups, indicating a subclinical elevation that may reflect ongoing but attenuated immune activation rather than overt autoimmune disease [71]. Among patients positive for anti-pulmonary autoantibodies, a marked female preponderance was observed alongside lower creatinine and troponin values — differences most plausibly attributable to sex-related confounding, given the well-established female predominance in autoimmune conditions, rather than to a specific disease mechanism. Anti-vascular autoantibody positivity showed no significant associations with the laboratory parameters examined. Combined tissue positivity by Western blot confirmed a statistically significant association with subclinical CRP elevation, reinforcing the pattern observed in the cardiac subgroup and further supporting the concept of an attenuated inflammatory state accompanying tissue-directed autoreactivity. The HEp-2 assay yielded a broader range of correlations — beyond CRP elevation, positivity was significantly associated with advancing age and female sex, in keeping with the well-documented increase in background autoreactivity and ANA prevalence with age and female biological sex in the general population [72]. Collectively, these laboratory findings suggest that tissue-specific autoreactivity in long COVID is accompanied by a subclinical inflammatory signature that may reflect genuine underlying immune activation yet falls below the detection threshold of routine clinical markers — underscoring both the subtlety of this immune phenotype and the need for more sensitive inflammatory profiling approaches in this patient population.

This study has several limitations that should be considered when interpreting the findings. The single-center, non-blinded design and reliance on self-reported symptoms introduce potential ascertainment bias and subjective variability in clinical characterization. The patient cohort was markedly heterogeneous with respect to age, sex, comorbidity burden, and the time elapsed since acute SARS-CoV-2 infection, all of which represent potential confounding variables that complicate the interpretation of serological profiles. Medication use was not systematically controlled, and given the overlap between comorbidities and their respective treatments, pharmacological confounding cannot be excluded. Taken together, these factors may limit the generalizability of our findings, particularly to patients with milder or less clinically characterized forms of long COVID.

The analysis of clinical and laboratory correlations was confined to the long COVID cohort, meaning that subgroup comparisons were made within the patient population rather than against a healthy or recovered control standard. This approach inherently limits the statistical power to identify robust, disease-specific markers and renders the detected associations more susceptible to within-group confounding. The observed correlations should therefore be interpreted with caution, as they may reflect relative differences between patient subgroups rather than true pathological distinctions from physiological baseline.

A further structural limitation concerns the imbalance between the patient and control cohorts, with 114 long COVID patients compared to 36 pre-pandemic controls. This disproportion reduces statistical power and increases the risk of both type I and II errors, particularly in tissue-specific comparisons such as vascular and pulmonary autoreactivity where effect sizes may be more modest. The longitudinal subsample available for repeated sampling was similarly limited in size, which constrains the conclusions that can be drawn regarding the temporal dynamics of autoantibody responses — including whether IgM persistence reflects ongoing immune activation or a gradual resolution that larger longitudinal cohorts might better capture. It should also be acknowledged that patient recruitment via general practitioner referral, based on clinically suspected long COVID symptoms, may introduce a degree of selection bias toward individuals with more prominent or persistent symptomatology. While this referral pathway captures a broader community-based population than tertiary care recruitment, patients with milder or subclinical forms of long COVID who did not seek or require medical attention remain underrepresented, potentially limiting the generalizability of the observed autoantibody prevalence estimates to the wider post-COVID population.

Perhaps the most significant limitation of this study is the absence of a comparator group comprising individuals who recovered from SARS-CoV-2 infection without developing long COVID. Although the use of pre-pandemic healthy controls permits the assessment of baseline physiological autoreactivity, it does not allow discrimination between immune alterations attributable to prior infection per se and those specifically associated with long COVID pathophysiology. Emerging evidence indicates that autoreactive signatures can be detected not only in long COVID but also in individuals who fully recover following acute infection, albeit often at lower magnitude or with qualitatively distinct patterns. Without an infection-matched recovered control group, it is therefore not possible to definitively attribute the observed tissue-specific autoreactivity to mechanisms unique to long COVID, as opposed to a broader post-viral immune remodeling process. This distinction carries important implications for causal inference and for the interpretation of the proposed pathophysiological model. Consequently, the high prevalence of tissue-specific autoantibodies observed in this cohort — particularly the IgM-dominant cardiac and vascular reactivity — cannot be unequivocally interpreted as long-COVID-specific. It remains plausible that a proportion of the detected autoreactivity reflects a post-viral immune imprint common to SARS-CoV-2 infection broadly, and that the differences observed relative to pre-pandemic controls overestimate the specificity of these findings to long COVID pathophysiology. The clinical correlations identified in this study, including those with cardiovascular and neurological symptom clusters, should therefore be interpreted within this context.

Closely related to this is the broader interpretive challenge of distinguishing association from causality. While our data demonstrate a clear association between tissue-specific autoantibody signatures and long COVID clinical phenotypes, they do not establish whether these autoreactive responses represent a primary pathogenic driver, a secondary epiphenomenon reflecting persistent tissue injury, or a component of a broader post-infectious immune recalibration that may occur even in clinically recovered individuals. Future studies incorporating longitudinal sampling across acute infection, uncomplicated recovery, and established long COVID will be essential to define the temporal and causal relationships between SARS-CoV-2 infection, tissue autoreactivity, and chronic symptom development.

Several methodological considerations also warrant acknowledgment. The Western blot assay employed in this study is inherently semi-quantitative; due to significant heterogeneity in band intensities, autoantibody levels could not be reliably quantified, limiting the analysis to a qualitative positive/negative assessment. Target antigen identification relied on approximate molecular weight estimation, which lacks the precision of sequence-based methods. The use of tissue homogenates introduces variability in protein abundance, precluding direct comparison with standardized quantitative platforms such as ELISA. Moreover, the denaturing conditions of SDS-PAGE expose linear neo-epitopes while potentially disrupting tertiary protein structures, meaning that antibodies directed against conformational epitopes may go undetected. The observed high background reactivity further highlights the difficulty of defining a clear diagnostic threshold under these conditions, as the transition from native to denatured states likely contributes to nonspecific signal. Regarding tissue source variability, cardiac and pulmonary homogenates were each prepared from a single donor specimen, while vascular homogenates were derived from pooled internal mammary artery segments obtained from multiple donors. All patient and control sera were tested against the same tissue preparations, ensuring internal consistency across comparisons. To assess inter-membrane reproducibility, a fixed positive control serum was applied to each Western blot membrane throughout the study. While this approach confirmed consistent band detection across membranes, it should be noted that the positive control serum was selected based on sample availability rather than formal analytical validation, which represents a limitation of the reproducibility assessment. The use of single-donor cardiac and pulmonary preparations, while ensuring homogeneity within the study, limits the generalizability of the antigenic targets identified, as donor-specific protein expression profiles may not fully represent the broader population-level tissue proteome. Validation across multiple independent donor preparations will be an important consideration for future studies aiming to confirm and extend these findings.

In conclusion, our study demonstrates that the majority of long COVID patients exhibit detectable tissue-specific autoantibodies, indicative of widespread underlying immunological dysregulation. This autoreactive signature is characterized by persistent IgM reactivity and heterogeneous isotype dynamics, with a particularly high prevalence of structural autoreactivity that remains undetected by standard serological screening. Importantly, these immune signatures carry clinical relevance: cardiac autoantibody positivity was associated with neurovascular manifestations, including hypertension and headache, while combined tissue autoreactivity correlated with persistent anosmia and ageusia. Collectively, these findings highlight the insufficiency of routine ANA HEp-2 testing in capturing this dimension of long COVID immune dysregulation, and suggest that tissue-based Western blot approaches may reveal a distinct autoreactive profile beyond the reach of conventional diagnostic tools.

Tissue-level autoreactivity may therefore represent a previously underrecognized immunological dimension of long COVID, with particular relevance to vascular and neurovascular dysfunction. The convergence of persistent IgM-dominant autoreactivity, tissue-specific immune targeting, and the emerging clinical correlations reported here supports the concept that long COVID may involve sustained immune–vascular crosstalk rather than isolated organ-specific injury. Future studies integrating antigen identification, comprehensive immune phenotyping, and functional vascular assessments will be essential to determine whether these autoreactive signatures constitute biomarkers of disease persistence, mechanistic contributors to chronic post-viral pathology, or both.

## Supplementary Information

Below is the link to the electronic supplementary material.

Supplementary file1. Schematic overview of the tissue-specific Western blot workflow. (A) Tissue Antigen Preparation & Quantification: Human Heart, Lung, and Internal Mammary Artery (IMA) samples are homogenized in SDS-PAGE buffer, boiled, centrifuged, and the protein concentration of the supernatant is quantified. (B) SDS-PAGE & Membrane Transfer: Tissue homogenates are separated via gel electrophoresis and transferred to nitrocellulose membranes, followed by blocking. (C) Imaging and Autoantibodies Detection: Membranes are cut into individual strips and incubated with patient serum (1:1000 dilution) followed by secondary antibodies (anti-human IgG/IgM). Strips are reassembled and imaged to detect specific autoantibody bands. Supplementary Fig. 2. Optimization of serum dilution for tissue-specific autoantibody detection. To determine the optimal serum dilution for maximizing detection of high-affinity, tissue-specific autoantibodies while minimizing nonspecific background reactivity, cardiac tissue homogenates were probed with a titration series of serum from a representative long COVID patient. Serum was tested at dilutions of 1:15, 1:30, 1:100, 1:300, 1:1,000, and 1:5,000, and incubated with either anti-human IgG-specific (left panel) or IgM-specific (right panel) secondary antibodies. Red arrows indicate the positions of the IgG and IgM heavy chains, which served as internal controls confirming consistent secondary antibody activity across all dilution levels. White arrowheads mark nonspecific low-affinity bands that progressively disappear with increasing dilution, while red arrowheads indicate high-affinity autoantibody bands persisting at higher dilutions. Based on these results, 1:1,000 was selected as the working dilution for all subsequent analyses. Supplementary Fig. 3. Determination of molecular weight and signal intensity. Representative images of Western blot strips showing the detection of IgM and IgG heavy and light chains alongside molecular weight standards. The lower panels display the software interface (GelAnalyzer 23.1.1) used for the quantitative assessment of protein bands. Intensity profiles were integrated to determine the area under the curve (AUC), and outlier analysis was applied to differentiate specific autoantibody signals from non-specific bands. Supplementary Fig. 4. Comprehensive map of autoantibody reactivities. A digital alignment of Western blot results showing the distribution of detected IgG (blue markers) and IgM (magenta markers) bands across the molecular weight spectrum for Heart, Artery, and Lung tissues. Each column represents an individual patient sample, illustrating the heterogeneity and lack of a single dominant antigenic target across the Long COVID cohort. (PDF 1566 kb)

## Data Availability

N/A.
