# Supplementary material for: Tissue-specific autoantibody signatures reveal immune alterations undetected by routine serology in long COVID
Source: GeroScience. 2026 May 12;48(3):3623–47. doi: 10.1007/s11357-026-02286-9 (PMC13356159; doi:10.1007/s11357-026-02286-9)

Supplementary figure 1

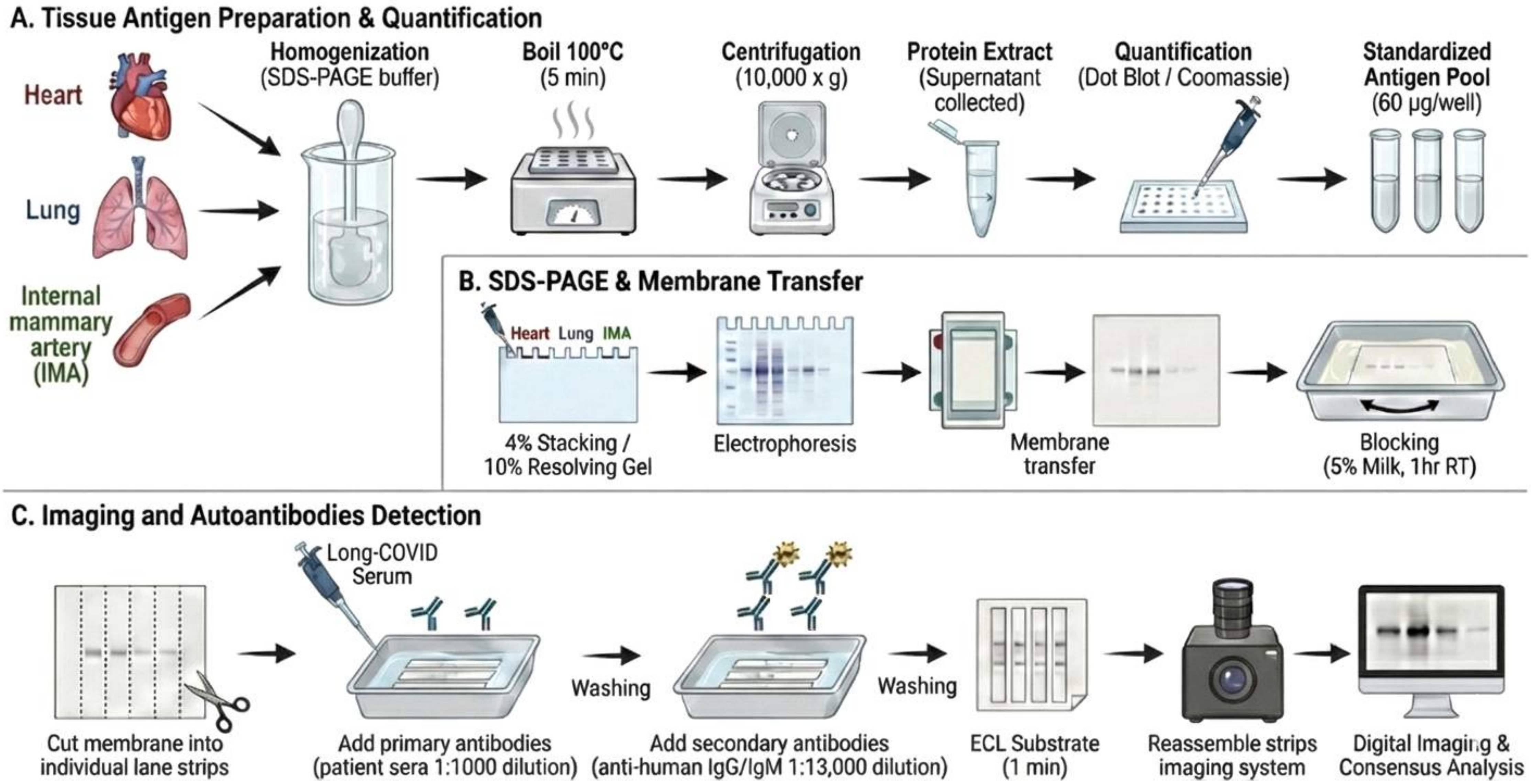

Supplementary figure 2

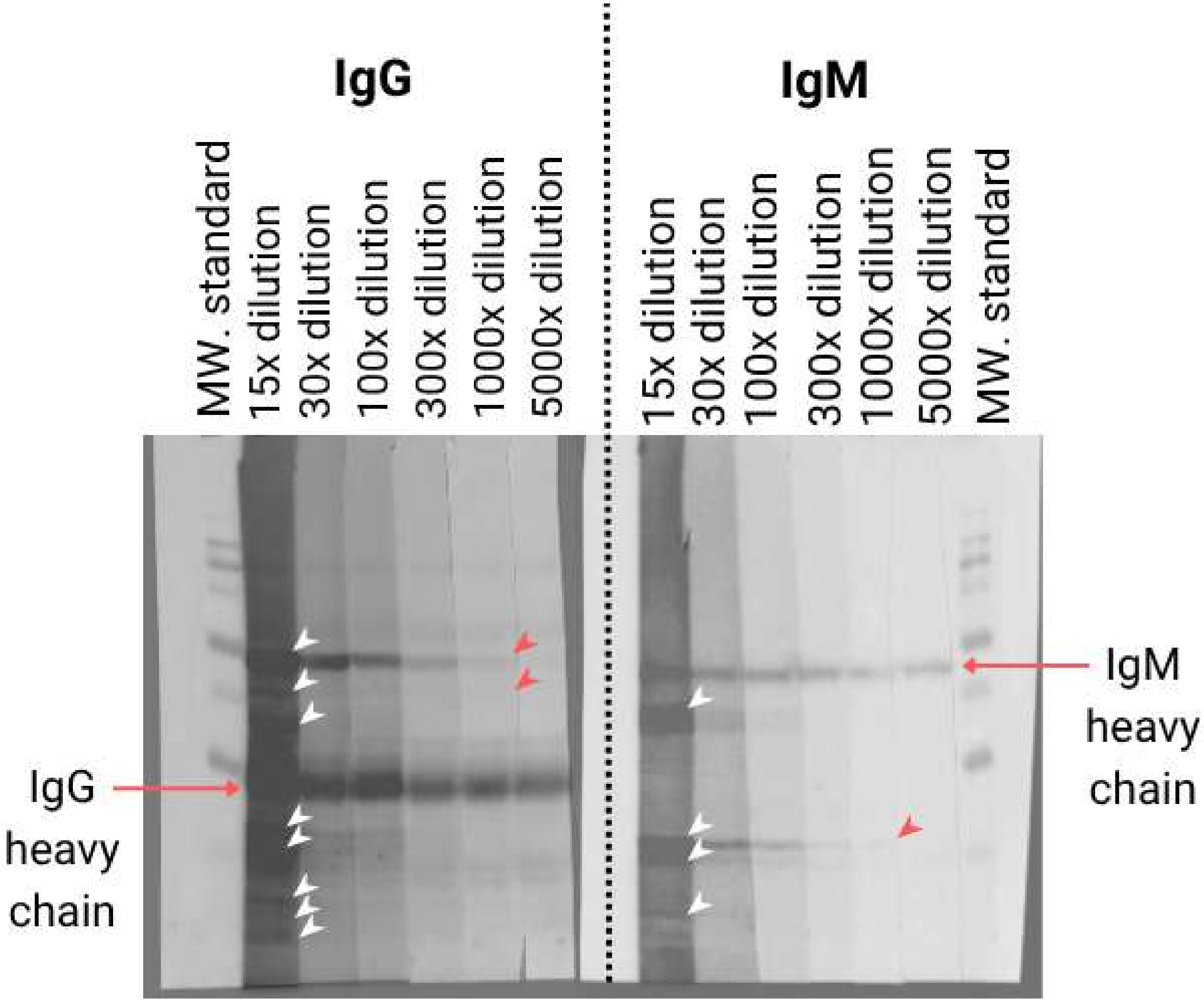

Supplementary figure 3

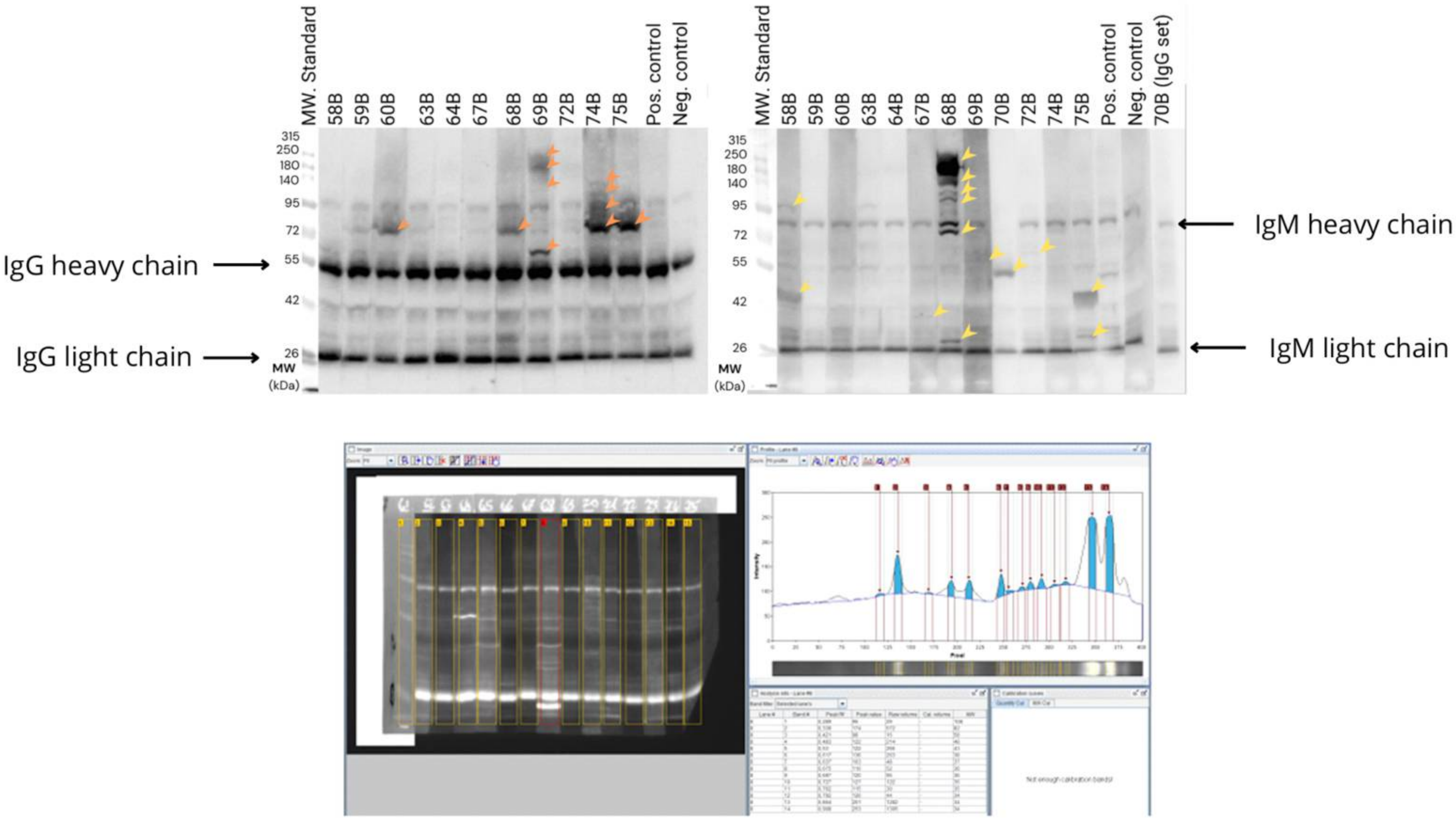

Supplementary 4

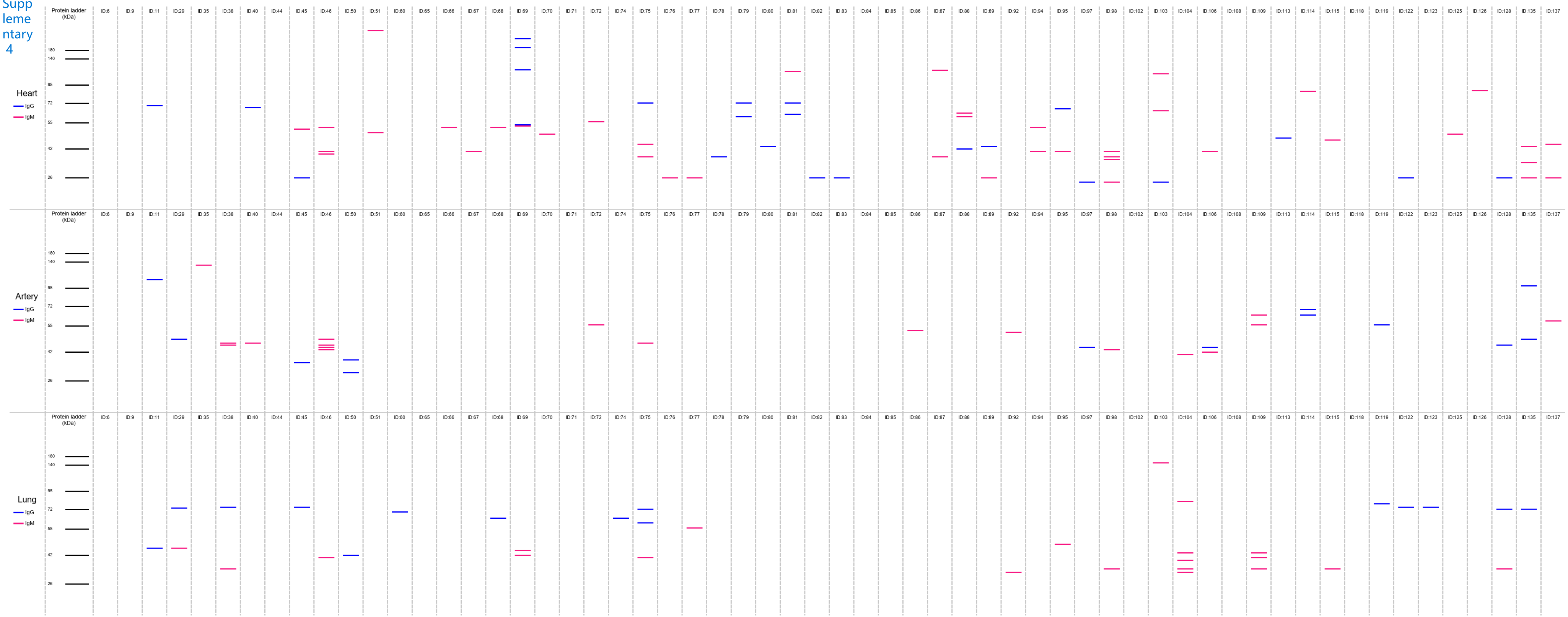

Supplement: Supplementary file 1 — Supplementary file1. Schematic overview of the tissue-specific Western blot workflow. (A) Tissue Antigen Preparation & Quantification: Human Heart, Lung, and Internal Mammary Artery (IMA) samples are homogenized in SDS-PAGE buffer, boiled, centrifuged, and the protein concentration of the supernatant is quantified. (B) SDS-PAGE & Membrane Transfer: Tissue homogenates are separated via gel electrophoresis and transferred to nitrocellulose membranes, followed by blocking. (C) Imaging and Autoantibodies Detection: Membranes are cut into individual strips and incubated with patient serum (1:1000 dilution) followed by secondary antibodies (anti-human IgG/IgM). Strips are reassembled and imaged to detect specific autoantibody bands. Supplementary Fig. 2. Optimization of serum dilution for tissue-specific autoantibody detection. To determine the optimal serum dilution for maximizing detection of high-affinity, tissue-specific autoantibodies while minimizing nonspecific background reactivity, cardiac tissue homogenates were probed with a titration series of serum from a representative long COVID patient. Serum was tested at dilutions of 1:15, 1:30, 1:100, 1:300, 1:1,000, and 1:5,000, and incubated with either anti-human IgG-specific (left panel) or IgM-specific (right panel) secondary antibodies. Red arrows indicate the positions of the IgG and IgM heavy chains, which served as internal controls confirming consistent secondary antibody activity across all dilution levels. White arrowheads mark nonspecific low-affinity bands that progressively disappear with increasing dilution, while red arrowheads indicate high-affinity autoantibody bands persisting at higher dilutions. Based on these results, 1:1,000 was selected as the working dilution for all subsequent analyses. Supplementary Fig. 3. Determination of molecular weight and signal intensity. Representative images of Western blot strips showing the detection of IgM and IgG heavy and light chains alongside molecular weight [file 11357_2026_2286_MOESM1_ESM.pdf]
